# Evolving CRBN ligands enhance the drug-like properties of protein degraders

**DOI:** 10.1080/14756366.2026.2700038

**Published:** 2026-07-08

**Authors:** Xin-Qian Ji, Lin Wang, Xing-Jie Dai

**Affiliations:** ^a^ State Key Laboratory of Metabolic Dysregulation & Prevention and Treatment of Esophageal Cancer, Key Laboratory of Advanced Drug Preparation Technologies, Ministry of Education of China, Key Laboratory of Henan Province for Drug Quality and Evaluation, Henan Province, School of Pharmaceutical Sciences, Zhengzhou University, Zhengzhou, China; ^b^ Xinyang Agriculture and Forestry University, Xinyang, China

**Keywords:** CRBN, PROTAC, molecular glue degrader, targeted protein degradation, drug-like properties

## Abstract

Proteolysis-targeting chimaeras (PROTACs) enable event-driven degradation of disease-relevant proteins by hijacking the ubiquitin–proteasome system (UPS). Cereblon (CRBN) is the most widely exploited recruiter due to its well-characterised binding pocket and strong clinical precedent from immunomodulatory imide drugs (IMiDs). However, early CRBN ligands have suboptimal pharmacokinetics, off-target neosubstrate degradation, immunomodulatory liabilities, and resistance mechanisms, which restrict the drug-like properties of CRBN-based degraders. This review systematically summarises CRBN ligand evolution and how chemical innovations improved PROTAC drug-likeness. We discuss classical IMiD-based ligands and their clinical translation, followed by next-generation recruiters, including non-IMiD scaffolds, conformationally constrained tricyclic and spirocyclic ligands, cyclic imides, covalent ligands, and stimuli-responsive designs. We highlight expanding application of novel CRBN ligands in molecular glue degrader development. Finally, key challenges and future directions for achieving selective, durable, and clinically viable CRBN-mediated protein degradation are discussed.

## Introduction

Over the past two decades, targeted protein degradation (TPD) has emerged as a promising therapeutic strategy, opening new avenues for drug development^1^. This approach leverages intracellular protein degradation pathways, including the autophagy-lysosome pathway and the UPS, to selectively eliminate disease-related proteins rather than merely inhibiting their activity^2^. In the UPS, substrates are tagged with ubiquitin through a multi-step enzymatic process involving E1 ubiquitin-activating enzymes, E2 ubiquitin-conjugating enzymes, and E3 ubiquitin ligases, which confer substrate specificity and catalyse the transfer of ubiquitin to defined lysine residues on target proteins. The resulting polyubiquitinated proteins are subsequently recognised and degraded by the 26S proteasome^3^. Harnessing this highly regulated system for therapeutic intervention has led to the development of PROTACs, representing one of the most influential innovations in the TPD field.

**Figure 1.**
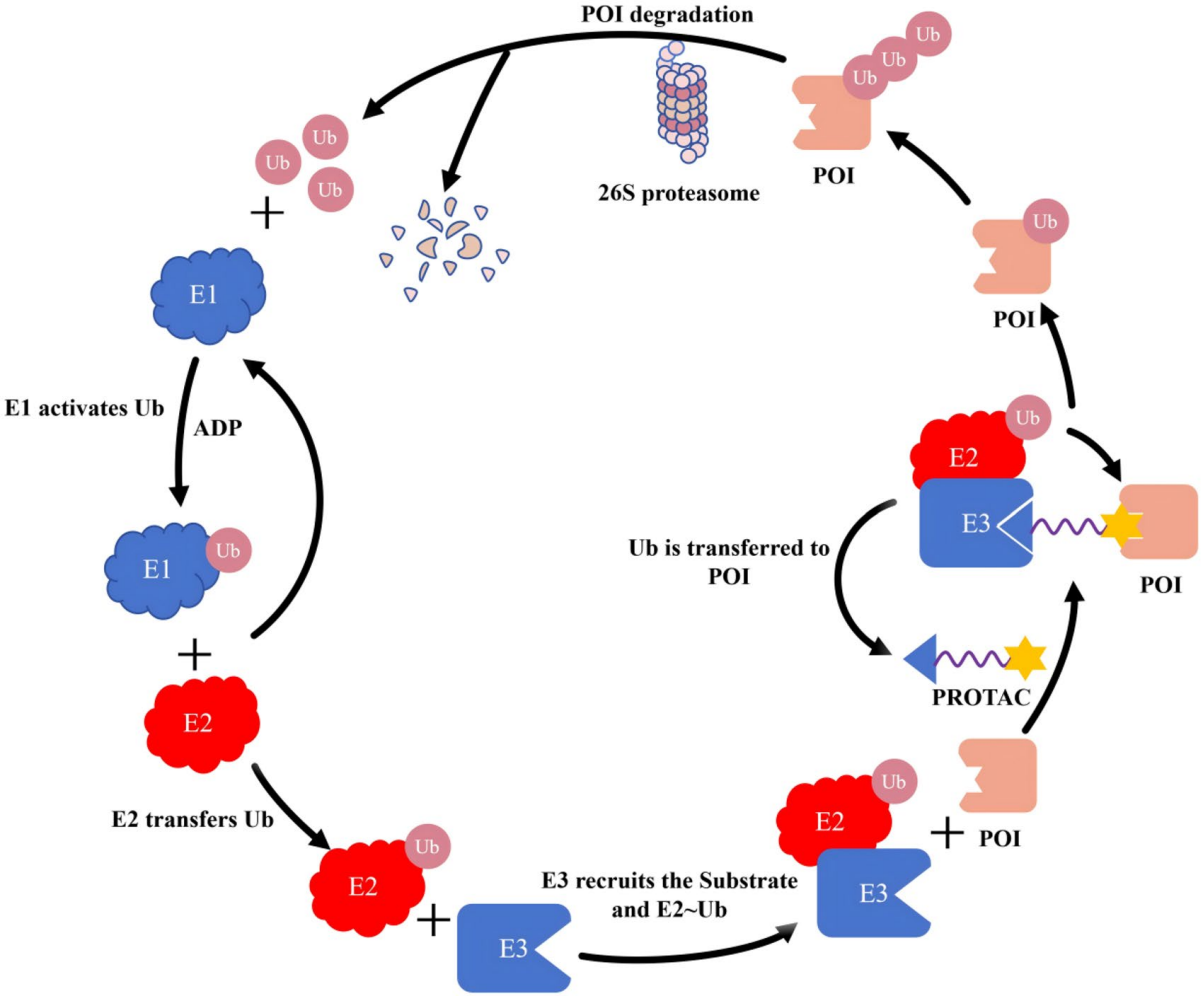
Mechanism of PROTAC-mediated POI degradation. Figure created using Microsoft PowerPoint software.

PROTACs are small bifunctional molecules composed of a protein of interest (POI) ligand and an E3 ubiquitin ligase ligand connected by an appropriate linker, enabling simultaneous engagement of both proteins and the formation of a productive ternary complex that drives ubiquitination and proteasomal degradation of the protein of interest (Figure 1)^4–6^. Compared with traditional small-molecule inhibitors, PROTACs offer several distinct advantages, including an event-driven and catalytic mode of action, the ability to target non-enzymatic or “undruggable” proteins, the potential to overcome resistance arising from target overexpression or mutation, and improved functional selectivity and safety profiles^7–9^.

Among the various E3 ligases employed in PROTAC design, CRBN is the most widely utilised recruiter due to its compact molecular size, well-characterised and chemically tractable binding pocket, and broad compatibility with diverse target ligands. CRBN-based PROTACs have demonstrated remarkable potential in both preclinical and clinical settings, particularly for targeting disease-driving proteins in cancer, autoimmune disorders, and neurodegenerative diseases^10–13^. Notably, candidates such as ARV-110, ARV-766 and ARV-471 have provided compelling validation CRBN-mediated degradation as a clinically viable therapeutically viable strategy.

However, despite these successes, CRBN ligands face significant challenges in clinical translation. These include suboptimal aqueous solubility, limited oral bioavailability, off-target neosubstrate degradation, and unfavourable pharmacokinetic profiles, which collectively restrict the therapeutic window of first-generation CRBN-based degraders^6^^,^^14–18^. To address these bottlenecks, extensive medicinal chemistry efforts have focused on the rational evolution of CRBN ligands beyond classical IMiD scaffolds, yielding next-generation chemotypes with refined selectivity, metabolic stability, oral bioavailability and decoupled neosubstrate activity.

Beyond the intrinsic chemical scaffold of CRBN ligands, the performance of CRBN-based PROTAC degraders is fundamentally modulated by ternary complex cooperativity, the conformational plasticity of CRBN, spatial characteristics of the linker moiety, and the overall efficiency of ubiquitination and proteasomal turnover^17^^,^^19–21^. The cooperative intermolecular recognition within the CRBN–PROTAC–POI ternary complex is central to achieving potent degradation activity and high target selectivity. Ligand binding can induce distinct conformational changes in CRBN, remodelling its substrate-binding interface and further influencing the recognition of both disease-related targets and endogenous neosubstrates. Differences in linker rigidity and flexibility also affect molecular spatial configuration and ternary complex assembly^22^. Meanwhile, the kinetics of ubiquitin transfer and subsequent proteasomal clearance serve as key mechanistic factors that profoundly shape the potency, selectivity, and clinical translational potential of CRBN-recruited protein degraders^23^.

This review systematically summarises the structural evolution and design strategies of modern CRBN ligands. We first overview classical IMiD-based recruiters and their clinical translational progress, followed by detailed discussion of emerging CRBN ligand classes, including non-IMiD scaffolds, conformationally constrained tricyclic/spirocyclic/fused heterocycles, cyclic imides, covalent ligands and stimuli-responsive analogs. We further highlight the expanding application of evolved CRBN chemotypes in the rational development of molecular glue degraders. Finally, key mechanistic challenges and future directions towards the design of selective, safe and clinically actionable CRBN-targeted protein degraders are discussed.

## IMiD-based CRBN ligands: clinical translation and intrinsic limitations

**Figure 2.**
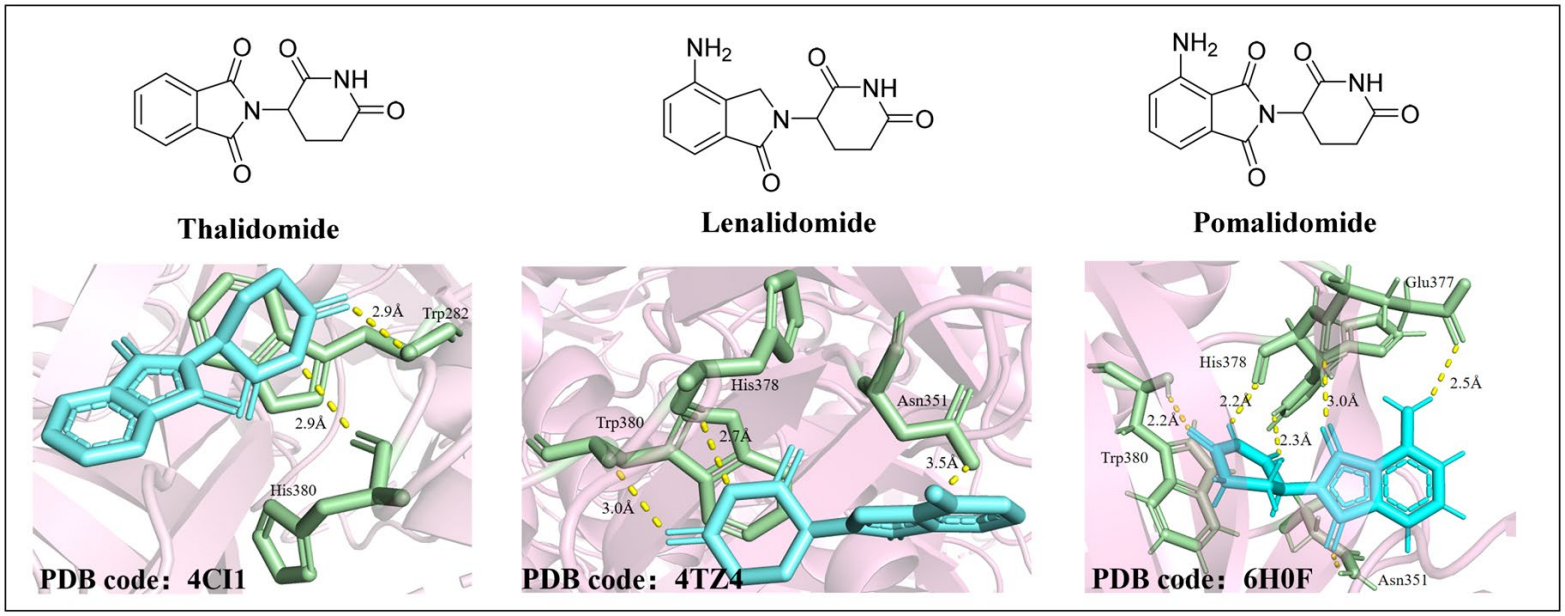
Representative IMiD-Based CRBN Ligands. Figure created using Pymol software.

Thalidomide and its structural analogues, lenalidomide and pomalidomide, collectively define the IMiDs class that constitutes the first-generation and most extensively used CRBN-binding ligands in PROTAC design (Figure 2)^24^. Structurally, thalidomide is composed of a glutarimide moiety fused to a phthalimide ring system, a configuration that underlies its high-affinity engagement with CRBN. Crystallographic and biochemical studies have established that the glutarimide ring is indispensable for anchoring the ligand within the hydrophobic binding pocket of CRBN, whereas the phthalimide/isoindolinone scaffold provides a chemically accessible exit vector for linker attachment in bifunctional degraders^25^.

Rational derivatization of the thalidomide scaffold has yielded second- and third-generation IMiDs with progressively improved pharmacological properties. Lenalidomide, which incorporates an amino substituent at the 4-position of the phthalimide ring, exhibits enhanced CRBN binding affinity and improved aqueous solubility relative to thalidomide^26^. Further modification led to pomalidomide, which introduces both a 4-amino group and a 5-carbonyl substitution, enabling a more favourable binding conformation within the CRBN pocket and translating into increased degradation potency and improved tolerability profiles^27^. Although these compounds were originally developed for distinct clinical indications, the discovery of their ability to hijack CRBN as a substrate receptor of the CRL4^CRBN^ E3 ubiquitin ligase complex fundamentally repositioned IMiDs as privileged recruiting elements in targeted protein degradation strategies^18^^,^^28^.

The clinical applications of CRBN-based PROTACs have expanded to a wide range of disease areas, particularly in the field of oncology^29^. For instance, ARV-393, currently in a phase I clinical trial (NCT06393738), is a PROTAC specifically designed to target B-cell lymphoma 6 (BCL6) (a transcription factor). It induces the degradation of the BCL6 protein, thereby blocking associated signalling pathways and inhibiting the growth of lymphoma cells^30^. Bruton’s tyrosine kinase (BTK)-targeting degraders, which show potential in the treatment of B-cell malignancies^31^^,^^32^. Furthermore, they have also provided therapeutic options for autoimmune diseases and neurodegenerative disorders. For example, an interleukin 1 receptor associated kinase 4 (IRAK4) degrader achieved an 85% improvement in skin lesions in an atopic dermatitis model. The development of PROTACs targeting Tau protein and α-synuclein is also underway, presenting new strategies for diseases such as Alzheimer’s disease. The widespread expression of CRBN across human tissues further supports the development of CRBN-based therapies for diverse organ systems^33^.

IMiD-based CRBN ligands have emerged as the mainstream choice for PROTAC development. As of 2025, among PROTAC molecules that have entered clinical stages globally, over 70% utilise CRBN as the E3 ligase, with the majority directly employing IMiDs or their derivatives as the CRBN-binding modules^16^.

Thalidomide, the prototypical CRBN ligand, suffers from suboptimal physicochemical and pharmacokinetic properties, including poor aqueous solubility and a complex metabolic profile, which contributed to its historical teratogenicity and limit its modern utility^34^^,^^35^. Mechanistically, it engages CRBN, altering the substrate specificity of the CRL4ᴬᴮ complex to promote the ubiquitination and proteasomal degradation of neosubstrates such as Ikaros zinc finger protein 1/3 (IKZF1/3)^36–39^. These limitations spurred extensive medicinal chemistry campaigns to optimise the thalidomide scaffold, aiming to improve potency, selectivity, and drug-like properties^35^.

Lenalidomide, a second-generation IMiD, incorporates rational structural modifications to enhance both efficacy and safety compared to its predecessor thalidomide^40^. It preserves high-affinity binding to CRBN while significantly improving the degradation of key lymphoid transcription factors, notably IKZF1/3, which play central roles in the pathogenesis of multiple myeloma (MM)^41^. Owing to its enhanced oral bioavailability and refined immunomodulatory profile, lenalidomide has become a cornerstone therapy in haematologic malignancies and is increasingly employed in autoimmune diseases. Its immunomodulatory activity – mediated in part by modulation of cytokine production – has broadened its therapeutic spectrum beyond oncology^42^^,^^43^.

Pomalidomide, a third-generation IMiD, represents a structurally refined derivative within the thalidomide-based scaffold series, demonstrating notable enhancements in both target affinity and proteasomal degradation dynamics^44^. Its improved pharmacological profile is characterised by an optimised balance between anti-myeloma potency and systemic tolerability. Moreover, pomalidomide exhibits superior efficacy in multiple myeloma models relative to its predecessors, and its molecular framework is particularly amenable to bifunctionalization, thereby serving as an ideal ligand for the development of PROTACs. These combined features underscore its dual utility as both a clinically effective therapeutic and a versatile chemical tool in targeted protein degradation strategies^38^^,^^45^.

ARV-110 is a PROTAC targeting AR, developed by Arvinas. It is currently in the final stage of phase II clinical trials (NCT03888662) and has demonstrated promising efficacy in clinical trials for the treatment of prostate cancer^11^^,^^46^. Its design incorporates a pomalidomide derivative as the CRBN ligand, and through optimisation of linker length and rigidity, it achieves highly efficient degradation of AR (DC_50_ ≈ 1 nM)^47^^,^^48^. Data from phase I/II clinical trials published in 2023 revealed that ARV-110 significantly reduced prostate-specific antigen (PSA) levels in patients with metastatic castration-resistant prostate cancer (mCRPC) who had undergone multiple lines of prior therapy, while exhibiting good tolerability. However, subsequent studies showed that it was unable to degrade the L702H point mutation and AR-V7 splice variant present in AR, leading to limitations in its efficacy^49^^,^^50^. Consequently, ARV‑766 has advanced as the lead candidate for subsequent clinical evaluation.

ARV-766 is an orally effective second-generation PROTAC degrader targeting AR, which incorporates three key structural modifications compared to ARV-110. First, the original six-membered ring has been replaced with a substituted four-membered ring, improving genotype coverage and potentially enabling potent degradation across multiple AR mutants. Second, a chlorine atom on the left-side aromatic ring has been substituted with a methoxy group, enhancing exposure under certain conditions. Third, the fluorine-substituted thalidomide moiety has been replaced by a fluorine-substituted benzamide in a single stereochemical configuration^51^. In cellular assays, ARV-766 effectively degrades wild-type AR in LNCap and VCaP prostate cancer cells, with DC_50_ values below 1.3 nM and approximately 1 nM, and D_max_ values exceeding 91% and 94%, respectively. It also demonstrates potent activity against various AR mutants, including L702H, while showing no detectable degradation of neosubstrates such as G1 to S phase transition 1 (GSPT1), IKZF1/3, or casein kinase 1 alpha (CK1α)^51^^,^^52^. *In vivo*, ARV-766 achieves significant tumour growth inhibition in mouse models. Clinically, phase I/II trial results reveal that ARV-766 offers improved efficacy and tolerability over ARV-110. A PSA50response was observed in 41% of patients with any AR ligand-binding domain (LBD) mutation and in 50% of those carrying the AR L702H mutation. Based on these promising outcomes, Arvinas plans to advance ARV-766 into phase III clinical trials.

ARV-471 is currently the most advanced PROTAC in clinical development, also developed by Arvinas for the treatment of ER-positive/**h**uman epidermal growth factor receptor 2 (HER2)-negative breast cancer, particularly in patients who have developed resistance to standard endocrine therapy^53^. Its CRBN ligand is optimised based on the lenalidomide structure^54^. ARV-471 has been recently approved by the FDA, and prior phase III clinical studies (NCT05654623) demonstrated high degradation efficiency, good oral bioavailability, and robust safety^55^. This approval represents a significant milestone in the clinical translation of targeted protein degradation.

In addition to Arvinas, multiple companies including Bristol Myers Squibb (BMS), Kymera, and Nurix have developed various IMiDs-based PROTACs that successfully target disease-associated proteins such as androgen receptor (AR), oestrogen receptor (ER), GSPT1, and IRAK4^16^. These agents span therapeutic areas including oncology, immunology, and neurodegenerative diseases (Table 1). For instance, NX-2127 (NCT04830137), a BTK-targeting PROTAC for B-cell malignancies. This molecule not only degrades canonical substrates like BTK but also non-canonical zinc finger proteins such as IKZF1/3, showcasing its versatility in targeted degradation and its potential to treat relapsed or refractory B-cell malignancies^49^^,^^64^^,^^65^. Similarly, KT-474 (NCT06083484) is an orally bioavailable PROTAC degrader targeting IRAK4 via recruitment of the E3 ligase CRBN, designed to overcome limitations of traditional kinase inhibitors by inducing both catalytic and scaffolding function inactivation through polyubiquitination-mediated proteasomal degradation^66^.

**Table 1.** Clinical CRBN-based PROTACs.

Company	Degrader	Target	Structure	The highest development phase	NCT number	Reference
Arvinas	ARV-110	AR	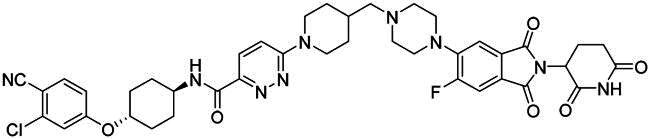	Phase II	NCT03888612	^56^
	ARV-766	AR	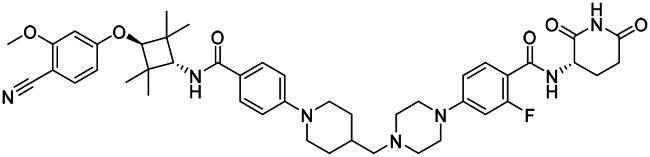	Phase II	NCT05067140	^56^
	ARV-471	ER	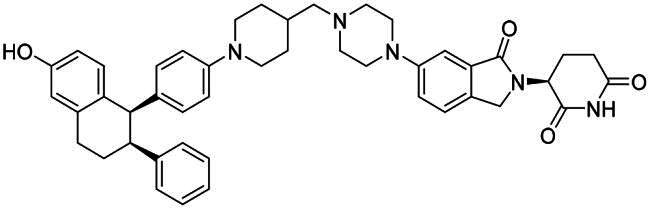	Phase III	NCT05654623	^57^
	ARV-393	BCL6	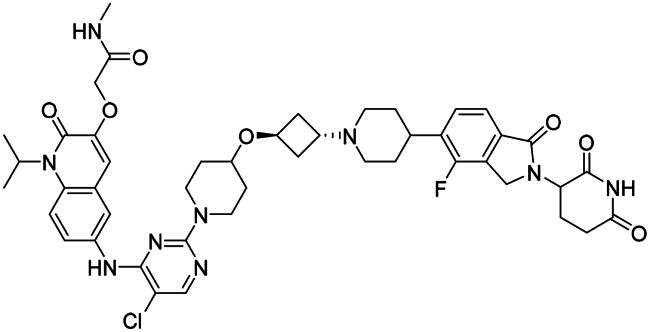	Phase I	NCT06393738	^58^
BMS	CC-94676	AR	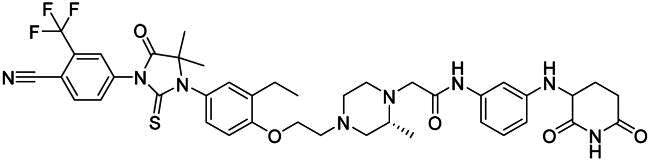	Phase I	NCT04428788	^59^
C4	CFT8634	BRD9	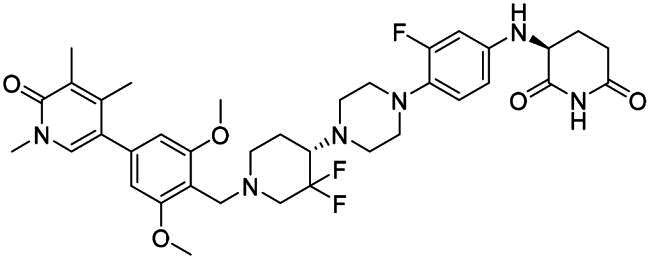	Phase II	NCT05355753	^2^ ^,^ ^60^
	CFT1946	BRAF V600E	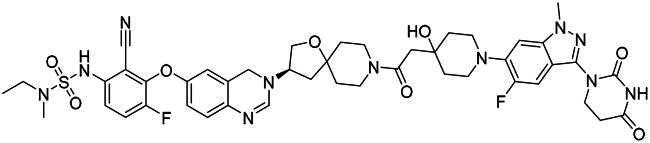	Phase II	NCT05668585	^11^ ^,^ ^60^
	CFT7455	IKZF1/3	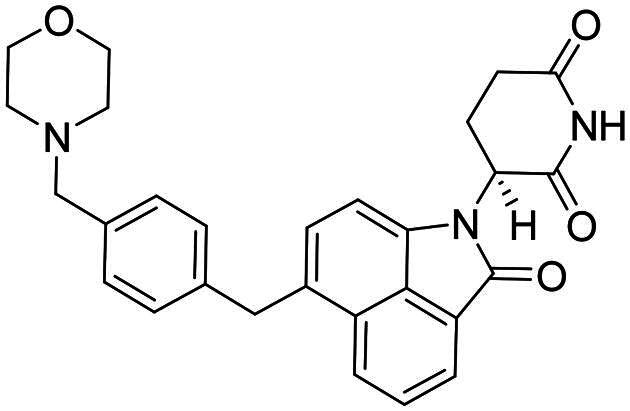	Phase II	NCT04756726	^61^
Kymera	KT-474	IRAK4	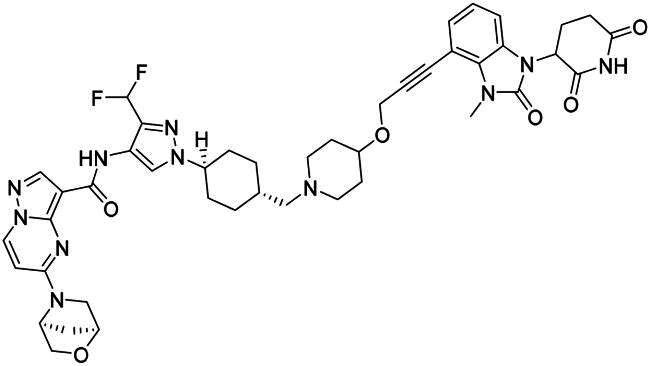	Phase II	NCT06083484	^56^
	KT-413	IRAK4	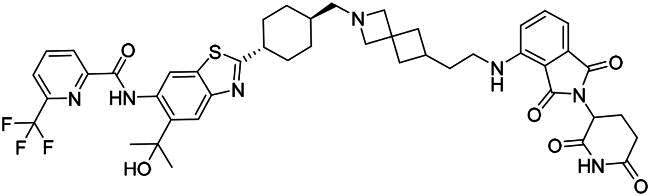	Phase I	NCT05233033	^2^
Nurix	NX-2127	BTK	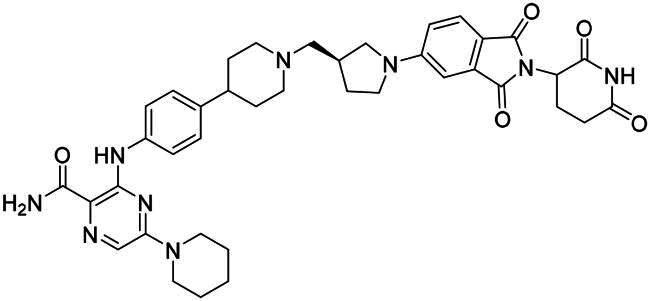	Phase I	NCT04830137	^56^
	NX-5948	BTK	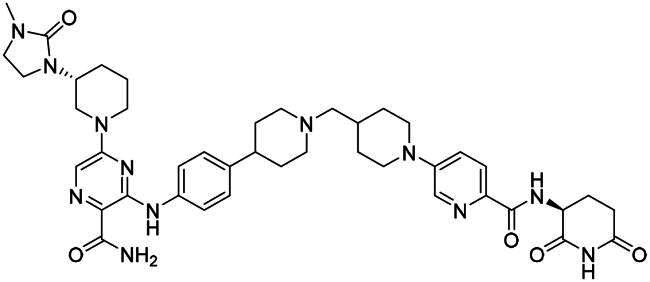	Phase I	NCT05131022	^56^
Accutar	AC682	ER	Structure undisclosed	Phase I	NCT05080842	^11^
	AC676	BTK	Structure undisclosed	Phase I	NCT05780034	^62^ ^,^ ^63^
Foghorm	FHD-609	BRD9	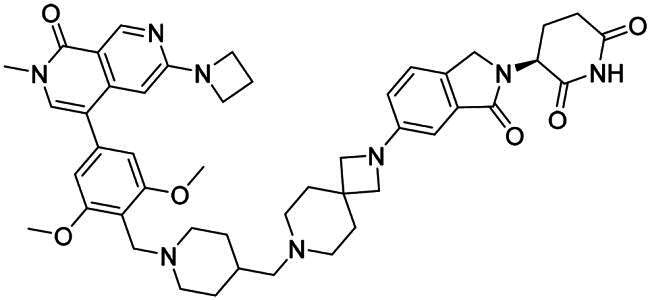	Phase I	NCT04965753	^56^
BeiGene	BGB16673	BTK	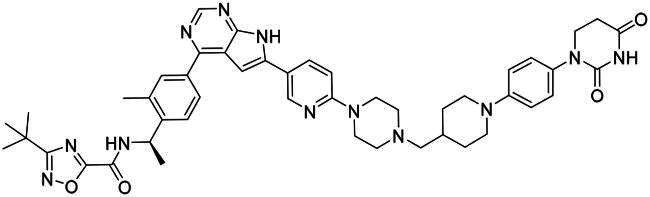	Phase I	NCT05006716	^11^
Haisco	HSK-29116	BTK	Structure undisclosed	Phase I	NCT04861779	^11^
Cullgen	CG001419	TRK	Structure undisclosed	Phase II	NCT03888612	^2^ ^,^ ^11^

Although clinical-stage CRBN-based PROTAC molecules (e.g. ARV-471, NX-2127) have shown potential in overcoming “undruggable” targets, their widespread clinical application remains constrained by key challenges^16^. These include suboptimal target specificity – exemplified by the off-target degradation of IKZF1/3 – compromised pharmacokinetic profiles, and the risk of drug resistance, such as that arising from neo-substrate competitive inhibition^16^^,^^67^. The molecular mechanisms driving PROTAC resistance can be categorised into four primary types. First, CRBN gene mutations occur at critical amino acid residues of CRBN; these mutations disrupt the normal binding of CRBN ligands, thereby impeding the formation of the ternary complex (CRBN-PROTAC-target protein)^68^. Second, target protein mutations – such as the L702H mutation in AR and the Y537S mutation in ER – alter the binding conformation between the target protein and the PROTAC, leading to reduced degradation efficiency^49^^,^^69^. Third, neo-substrate competitive inhibition takes place when certain intracellular proteins compete with the target protein for binding to the CRBN-PROTAC complex, diminishing the degradation of the intended target^70^. Fourth, dysfunction of the UPS – including reduced activity of E2 ubiquitin-conjugating enzymes and inhibition of proteasome activity – prevents the effective degradation of ubiquitinated target proteins^71^. To tackle these drug resistance issues, current research has proposed several counterstrategies. These include developing multi-target PROTACs (which simultaneously target the primary disease-associated protein and resistance-related proteins), optimising the structure of CRBN ligands to accommodate mutant CRBN, and combining PROTACs with UPS activators. For example, ARV-766 – through structural refinements to both its CRBN ligand and AR-binding domain – successfully addresses the limitation of ARV-110, which fails to degrade the AR L702H mutant^52^. Additionally, preliminary *in vitro* studies have explored combining PROTACs with proteasome activators, and this approach has been shown to significantly enhance the degradation efficiency of target proteins in drug-resistant cells^72^.

Despite the widespread application of IMiD-derived CRBN ligands and their clinically validated PROTAC candidates, this class of scaffolds still bears multiple inherent drawbacks and safety concerns^73^^,^^74^. Beyond suboptimal physicochemical and pharmacokinetic properties, conventional IMiD-based CRBN engagement frequently triggers aberrant degradation of physiological neo-substrates including IKZF1/3, GSPT1, CK1α and Sal-like protein 4 (SALL4), thereby disrupting haematopoietic homeostasis, cell cycle regulation and embryonic developmental signalling^40^^,^^74^^,^^75^. In addition, their intrinsic immunomodulatory properties may disturb immune balance and raise adverse reaction risks, while the thalidomide scaffold itself still carries a residual teratogenic risk^76^^,^^77^. Furthermore, drug resistance driven by CRBN mutations, target protein alterations, neo-substrate competitive interference and ubiquitin–proteasome system dysfunction further limits long-term clinical efficacy^78^. Collectively, these pharmacokinetic deficiencies, off-target liabilities, immunological risks and drug resistance bottlenecks strongly motivate the rational design and structural innovation of next-generation non-IMiD CRBN ligands with improved selectivity, drug-like properties and clinical applicability.

## Next-generation CRBN ligands: structural innovations and design strategies

**Figure 3.**
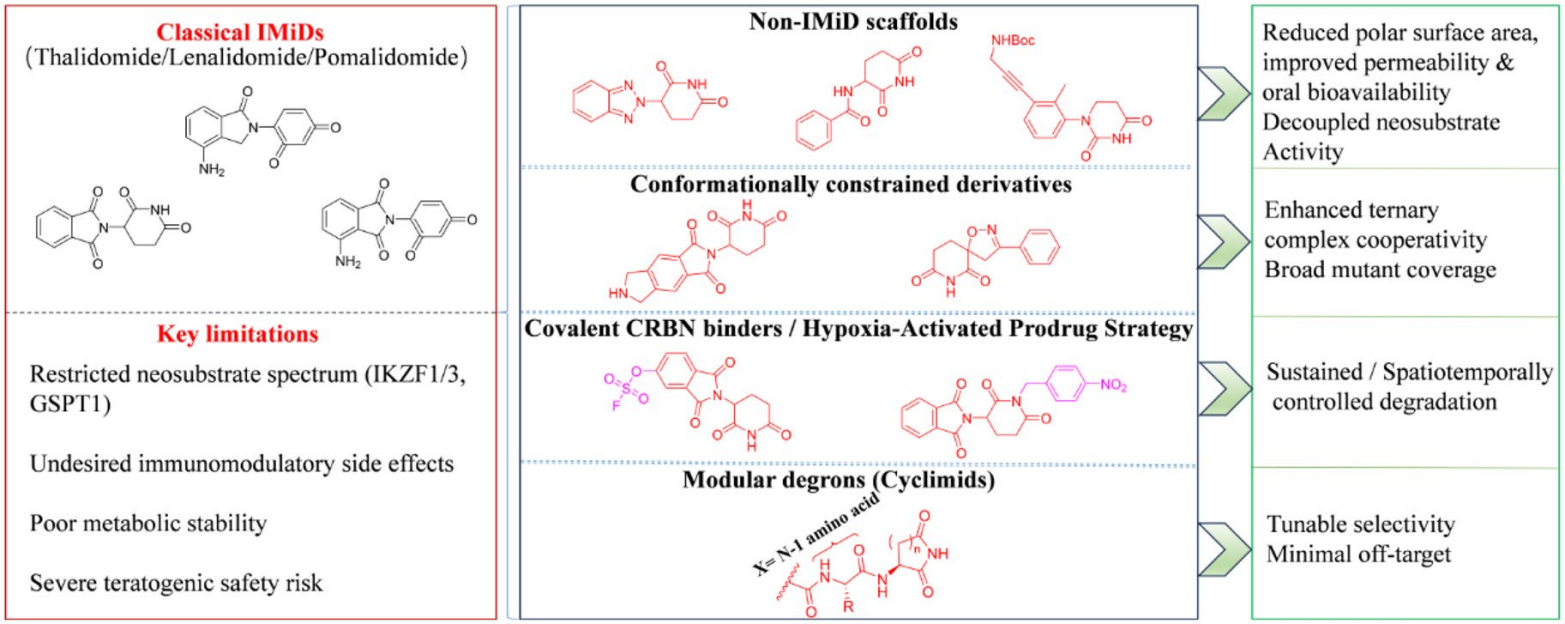
Next-Generation CRBN Ligands: Structural Innovations and Design Strategies. Figure created using Microsoft PowerPoint software.

Currently, PROTAC design employs various CRBN ligands like Lenalidomide, Thalidomide, and Pomalidomide^79^. These compounds face issues like racemisation, metabolic instability, immunogenicity, and poor pharmacokinetic profiles which have driven the development of next-generation non-IMiD CRBN recruiters^79^^,^^80^. Researchers are developing new CRBN ligands to overcome PROTAC’s off-target, suboptimal drug-like properties, and enhance pharmacokinetics and safety (Figure 3)^16^^,^^81^. Over the past two decades, beyond modifying thalidomide/lenalidomide/pomalidomide cores, researchers introduced cyclic/spirocyclic structures and substituent functional groups to regulate binding affinity, improve metabolic stability, drug selectivity, and optimise oral absorption^82^^,^^83^ Chemical modifications broaden CRBN ligands’ application in PROTAC, targeting tumour-related proteins and offering new treatments for autoimmune diseases^12^^,^^84^.

### Non-IMiD scaffolds

To overcome the intrinsic limitations of classical IMiD-based CRBN ligands – including inefficient polarity utilisation, intrinsic molecular glue activity, and limited control over degradation selectivity – a growing body of work has focused on the development of non-IMiD CRBN recruiters^85^. Despite structural diversity, these ligands can be broadly categorised into two complementary design strategies: compact heteroaromatic scaffolds that prioritise ligand efficiency and drug-like properties, and programmable non-IMiD scaffolds designed to decouple CRBN recruitment from neo-substrate degradation and enable predictable ternary complex formation^79^^,^^86^. Together, these approaches highlight a paradigm shift from IMiD mimicry towards rational engineering of CRBN recruiter behaviour.

#### Benzotriazole and related scaffolds

One major design strategy for non-IMiD CRBN ligands focuses on improving ligand efficiency and pharmacokinetic properties by replacing the canonical phthalimide scaffold with compact heteroaromatic systems^85^. Typically composed of a phenyl ring fused to an azole moiety or dual nitrogen-containing heterocycles, these ligands were developed to address the polarity inefficiency and limited chemical space associated with IMiDs^86^. By maximising productive heteroatom engagement while minimising redundant polar functionality, this class of ligands enables the construction of more drug-like CRBN-based degraders. Representative examples of such compact heteroaromatic CRBN ligands are summarised below.

**Figure 4.**
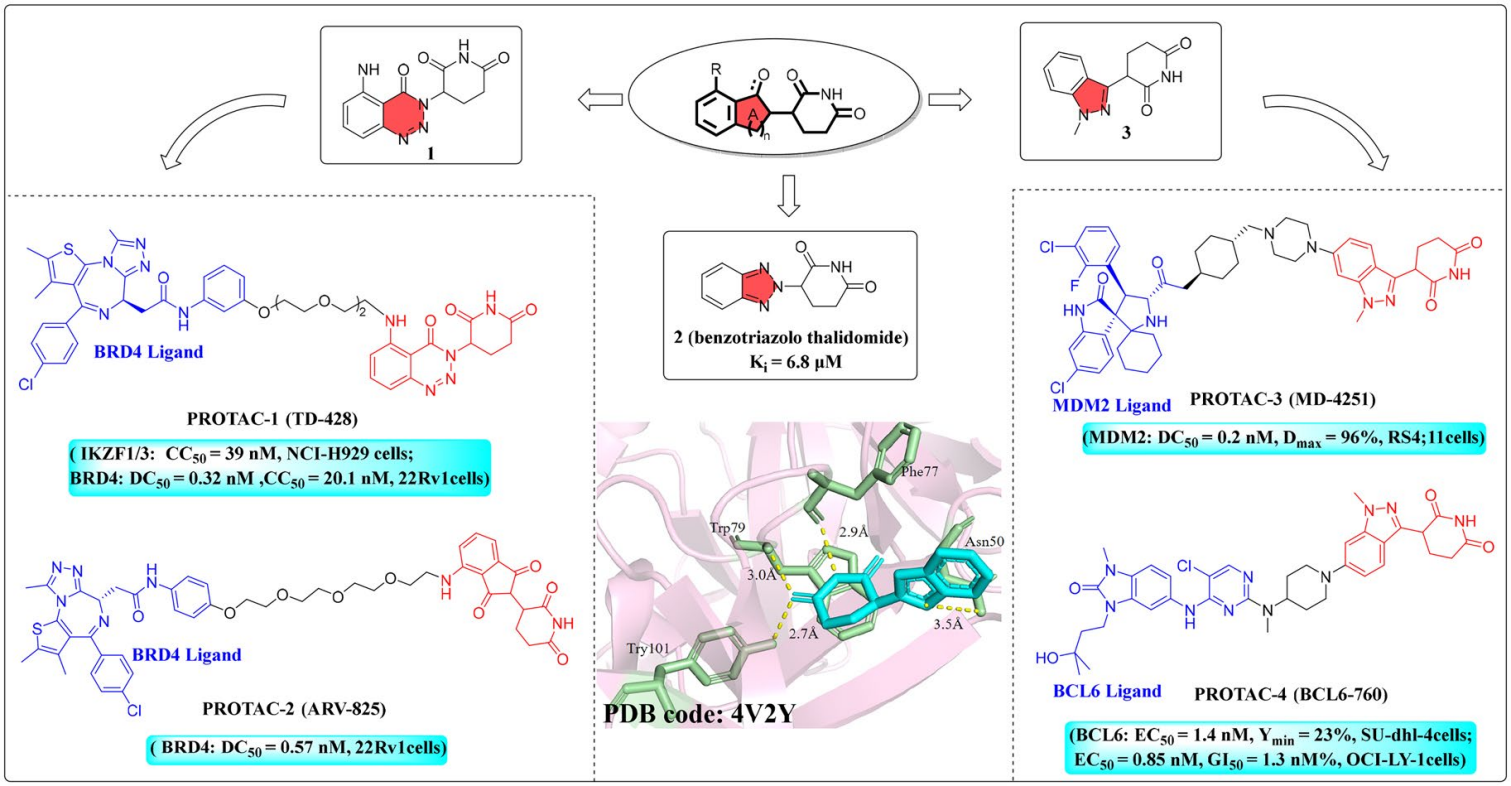
PROTACs based on benzotriazole and related scaffolds as CRBN ligands. These scaffolds were designed to replace the phthalimide portion of classical IMiD-based CRBN ligands with more compact heteroaromatic systems, aiming to improve ligand efficiency, reduce redundant polarity, enhance cellular permeability, and decrease off-target neosubstrate degradation. Figure created using Pymol and Chemdraw20.0 software.

Kim et al. replaced the phthalimide portion of classical IMiD-based CRBN ligands with an aminobenzotriazino scaffold (compound 1), which effectively degraded IKZF1/3 (CC_50_ = 39 nM) in NCI-H929 myeloma cells (Figure 4)^87^. PROTAC-1 (TD-428) was constructed by linking compound 1 to JQ1 via a meta-substituted phenyl linker, showed potent bromodomain-containing protein 4 (BRD4) degradation (DC_50_ = 0.32 nM) and strong antiproliferative activity in 22Rv1 (Human prostate cancer cells) (CC_50_ = 20.1 nM), surpassing PROTAC-2 (ARV-825). The enhanced potency is likely attributed to the potentially reduced polar surface area of the benzotriazole core, which may improve cellular permeability, and to the optimal ternary complex geometry conferred by the meta-substituted linker. In addition, PROTAC-1 exhibited approximately 100-fold lower off-target degradation of IKZF1/3 compared to ARV-825 and no hook effect, likely due to the unique binding mode of the aminobenzotriazino scaffold within CRBN.

Krasavin et al. designed a novel thalidomide analogue in which the phthalimide core was replaced by a benzotriazole ring, aiming to eliminate the unsatisfied polar carbonyl group while retaining CRBN-binding capability (Figure 4)^88^. The resulting compound 2 (“benzotriazolo thalidomide”) was synthesised via a Rh (II)-catalyzed N–H insertion using a diazocarbonyl intermediate, achieving high regioselectivity. Compared to thalidomide, compound 1 exhibited improved CRBN-binding affinity (*K*_i_ = 6.8 µM vs. 8.5 µM). X-ray crystallography revealed a binding pose analogous to thalidomide, with retention of key glutarimide-mediated interactions but loss of hydrogen bonding from the phthalimide carbonyl. *In vitro*, compound 2 displayed no significant cytotoxicity against MOLP-8 and KMS-12-PE myeloma cell lines up to 250 µM, and induced lower levels of late apoptosis than thalidomide at 300 µM.

Acharyya et al. reported the discovery of PROTAC-3 (MD-4251), a first-in-class orally bioavailable mouse double minute 2 (MDM2) degrader (Figure 4)^89^. Its CRBN ligand compound 3 was derived through structural optimisation of the 1-methyl-1H-indazole moiety used in CFT-1946 (Table 1), combined with systematic optimisation of the linker. Replacement of an amide with an amine in the linker, and substitution of piperidine with piperazine collectively improved oral bioavailability to 39% in mice. In RS4;11 leukaemia cells, PROTAC-3 achieved potent and rapid MDM2 degradation (DC_50_ = 0.2 nM; D_max_ = 96% at 2 h). PROTAC-3 induced sustained MDM2 depletion and p53 activation. Notably, a single oral dose of 50 mg/kg induced complete and durable tumour regression without toxicity, highlighting the therapeutic advantage of sustained MDM2 depletion over conventional inhibitors.

Shunatona et al. reported the discovery of PROTAC-4 (BCL6-760), a highly efficient BCL6 degrader (Figure 4)^90^. The molecule features a CRBN ligand derived from a 6-aminoindazole scaffold – the same CRBN-binding moiety utilised in PROTAC-3 – conjugated via an aminopiperidine linker to a BCL6-targeting ligand bearing a solubilising 2-methylbutan-2-ol side chain, contributing to its high potency (EC_50_ = 0.85–1 nM) and measurable oral bioavailability (*F* = 22%). PROTAC-4 did not degrade canonical CRBN neosubstrates, indicating that the 6-aminoindazole scaffold presents a binding surface less permissive for off-target recruitment. In an OCI-LY1 xenograft model, it achieved >90% BCL6 suppression and 64% tumour regression at 60 mg/kg BID, demonstrating its promise as an orally available BCL6 degrader.

#### Benzamide, phenyl dihydrouracil (PDHU), and related non-IMiD CRBN ligands

In contrast to ligand efficiency–driven designs, a second class of non-IMiD CRBN ligands was primarily developed to overcome the intrinsic molecular glue activity of IMiDs and the associated risk of unintended neo-substrate degradation. Scaffolds based on benzamide, PDHU, and related frameworks provide a more neutral and programmable CRBN interaction interface, allowing CRBN recruitment to be decoupled from IKZF1/3 or GSPT1 degradation^91^. These ligands enable finer control over ternary complex geometry and degradation selectivity, thereby improving the predictability and safety of CRBN-based PROTACs. Key examples of this programmable recruiter strategy are discussed below.

**Figure 5.**
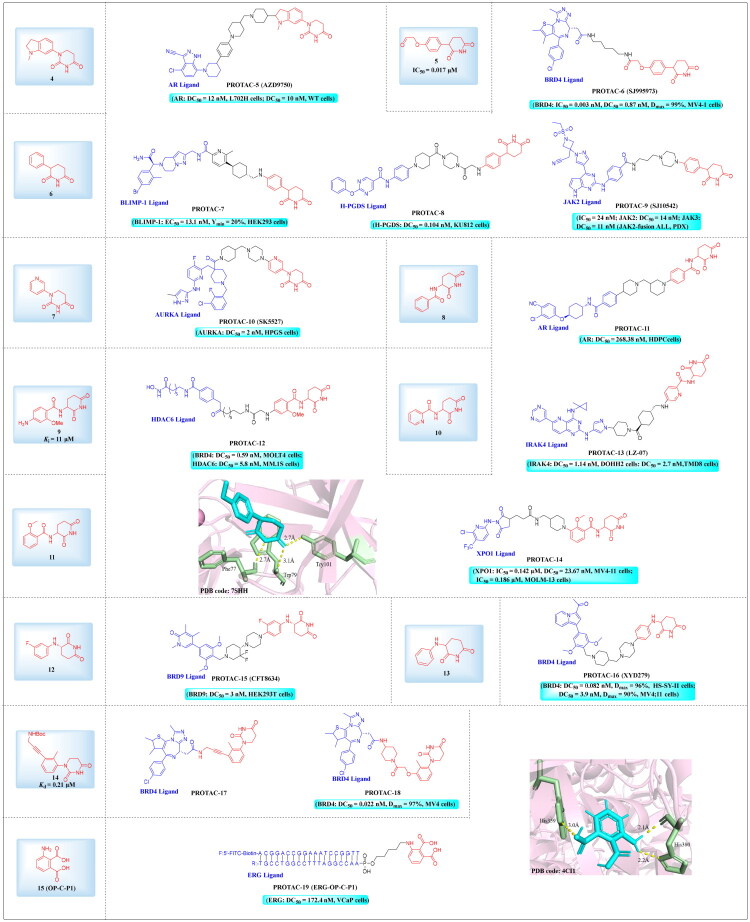
PROTACs based on benzamide, PDHU, and related scaffolds. These programmable non-IMiD scaffolds were developed to decouple CRBN recruitment from intrinsic IMiD-like molecular glue activity, thereby improving chemical stability, reducing undesired IKZF1/3 or GSPT1 degradation, and enabling finer control over ternary complex geometry, selectivity, and oral exposure. Figure created using Pymol and Chemdraw20.0 software.

Scott et al. identified a cyanoindole lead compound through high-throughput screening and successfully developed an orally bioavailable AR PROTAC degrader, PROTAC-5 (AZD9750), by employing computational modelling and structure-based optimisation strategies (Figure 5)^92^. This molecule recruits the CRBN E3 ligase via a dihydrouracil (DHU) moiety (compound 4), enabling potent degradation of both wild-type and L702H-mutant AR. A key breakthrough was the mitigation of mitotoxicity observed in earlier analogs through stereochemical optimisation (adopting a 3S-piperidine configuration), while avoiding off-target degradation of proteins such as Ikaros and Aiolos. In both seminal vesicle involution assays and the ST1273 patient-derived xenograft (PDX) model, PROTAC-5 demonstrated dose-dependent suppression of AR signalling and significant antitumor efficacy (85% tumour growth inhibition). Although residing in beyond-Rule-of-5 chemical space, the compound exhibited favourable oral bioavailability across multiple species (34% in rat, 47% in mouse, 40% in dog), positioning it as a novel oral degrader candidate with a differentiated safety profile for the treatment of castration-resistant prostate cancer.

Min et al. designed phenyl glutarimide (PG) derivatives as chemically stable CRBN-binding alternatives to IMiDs (Figure 5)^93^. Replacing the hydrolysis-prone phthalimide with a phenyl ring improved chemical stability (t_1/2_ >24 h vs. 4.2 h for IMiD-based dBET1) while retaining high CRBN affinity (IC_50_ = 0.017 μM). The lead PROTAC-6 (SJ995973) achieved potent BRD4 degradation (DC_50_ = 0.87 nM) and exceptionally strong antiproliferative activity in MV4-11 cells (IC_50_ = 0.003 nM), outperforming dBET1 by >2000-fold. PROTAC-6 did not degrade canonical CRBN neosubstrates (IKZF1, CK1α), indicating favourable selectivity. Mechanistic studies confirmed CRBN- and proteasome-dependent degradation. These findings establish PG as a valuable CRBN-binding scaffold that combines chemical stability, high ligand efficiency, and potent degradation activity.

Simmons et al. report the discovery and optimisation of PROTAC-7, the first orally bioavailable heterobifunctional degrader targeting B-lymphocyte-induced maturation protein-1 (BLIMP-1) (Figure 5)^94^. This campaign employed a well-validated CRBN-binding moiety (derived from compound 6) with balanced degradation efficacy, selectivity, and physicochemical properties as the foundation for optimisation. Replacing the imidazole ring in the target-binding moiety with a less basic pyrazole maintained nanomolar-level degradation potency (EC_50_ = 13.1 nM, Ymin = 20%) in HEK293 kidney cells while significantly reducing the Caco-2 efflux ratio from 8.2 to 3.7 and improving metabolic stability. Subsequent incorporation of a 6-methylpyridine ring into the linker further reduced the efflux ratio to 3.6 without compromising degradation activity. The resulting PROTAC-7 demonstrated selective degradation of the BLIMP-1α isoform, achieved approximately 60% sustained target degradation (over 14 h) following oral administration (200 mg/kg BID) in MM1.s xenograft models, and exhibited 18.8% oral bioavailability. The CRBN-dependent mechanism was validated by complete abolition of anti-proliferative effects with the CRBN inhibitor CBM-4077, successfully transforming an *in vitro* degradation tool into an orally available *in vivo* probe.

Osawa et al. designed novel haematopoietic prostaglandin D synthase (H-PGDS)targeting PROTACs by incorporating PG (compound 6) as CRBN ligand to improve chemical stability and metabolic resistance (Figure 5)^95^. The PROTAC-8 induced potent H-PGDS degradation in KU812 leukaemia cells, with DC_50_ values of 0.104 nM comparable to the previously reported PROTAC (DC_50_ = 0.036 nM).

Alcock et al. reported the design of PG-based PROTACs targeting Janus kinase 2/3 (JAK2/3), aiming to reduce off-target GSPT1 degradation associated with IMiD-based scaffolds (Figure 5)^96^. Using baricitinib- and ruxolitinib-derived warheads, a series of PG–PROTACs were synthesised. Lead compound PROTAC-9 (SJ10542) demonstrated potent and balanced degradation of JAK2 and JAK3 in a PDX model SIBALL020589, with DC_50_ values of 14 nM and 11 nM, respectively, while markedly sparing GSPT1. The degradation was confirmed to be CRBN-dependent, and PROTAC-9 exhibited potent cytotoxicity (IC_50_ = 24 nM) across multiple JAK-STAT-driven disease models. This study establishes PROTAC-9 as a highly efficient and selective JAK2/3 degrader, providing a valuable chemical tool for investigating the biological functions of JAK2/3 degradation. CRBN dependency was confirmed in knockdown assays, and no hook effect was observed at effective concentrations.

Krols et al. report the discovery and optimisation of PROTAC-10 (SK5527), a potent, selective, and intravenously administrable heterobifunctional degrader of AURKA (Figure 5)^97^. Initial structure–activity relationship efforts focused on replacing the thalidomide-based CRBN-binding moiety with arylaminoglutarimide and dihydrouracil scaffolds (compound 7). This modification aimed to address stability and racemisation issues associated with thalidomide, while improving physicochemical properties through elimination of a stereocenter and a hydrogen bond donor, thereby potentially enhancing membrane permeability. Subsequent linker rigidification via incorporation of piperazine and azetidine spacers significantly improved metabolic stability and plasma half-life. PROTAC-10, demonstrated potent AURKA degradation (DC_50_ = 2 nM) in MYCN-amplified neuroblastoma cells, accompanied by MYCN destabilisation. The degradation mechanism was confirmed to be CRBN- and proteasome-dependent. Kinome-wide selectivity profiling revealed >2000-fold selectivity for AURKA over AURKB, and at 100 nM, PROTAC-10 engaged significantly only with AURKA and TrkA among 403 non-mutant kinases. Pharmacokinetic properties were markedly improved, with intravenous clearance reduced to 12 ml/min/kg. A single intravenous dose (15 mg/kg) effectively reduced AURKA protein levels in tumour tissues *in vivo*.

Mao et al. developed PROTAC-11, a novel AR-targeting PROTAC optimised for non-invasive topical treatment of androgenetic alopecia (AGA), by employing a benzamide analog-based CRBN ligand (compound 8) with lower calculated logarithm of the partition coefficient (clogP) and topological polar surface area (tPSA) values than thalidomide to enhance skin penetration and retention (Figure 5)^98^. Structure-activity studies revealed that a piperidine-based linker combined with this CRBN ligand conferred superior skin retention (2.48%) and potent AR degradation (DC_50_ = 268.38 nM) in human dermal papilla cells (HDPCs). Topical application of PROTAC-11 in mice demonstrated high dermal accumulation, minimal systemic exposure, and significant hair regrowth comparable to 5% minoxidil, while *in vitro* and *in vivo* analyses confirmed its ability to downregulate AR and transforming growth factor-beta 1 (TGF-β1) while upregulating β-catenin, highlighting its potential as a targeted AGA therapy.

Steinebach et al. developed a novel class of nonphthalimide benzamide-type CRBN ligands by introducing ortho-substituents such as fluorine and methoxy groups to enhance CRBN binding affinity and improve conformational rigidity through intramolecular hydrogen bonding (Figure 5)^85^. Representative compounds (e.g. compound 9) exhibited favourable stability and reduced neosubstrate degradation. These ligands were further incorporated into BRD4- and histone deacetylase 6 (HDAC6) targeting PROTACs (e.g. PROTAC-12), achieving potent degradation activities (BRD4: DC_50_ = 0.59 nM; HDAC6: DC_50_ = 5.8 nM), outperforming benchmark degraders such as dBET57 and A6. Importantly, these benzamide-based PROTACs did not induce degradation of IMiD-associated neosubstrates, demonstrating their potential as selective and safer therapeutic agents.

Li et al. developed IRAK4-targeting PROTACs using simplified benzamide-type CRBN ligands (compound 10) as alternatives to IMiDs to reduce off-target IKZF1/3 degradation (Figure 5)^99^. Through systematic optimisation of linker rigidity (moderate rigidity preferred; meta-position > ortho-substitution) and the aromatic ring of the CRBN ligand (pyridine > benzene > pyridazine), the authors identified PROTAC-13 (LZ-07). PROTAC-13 exhibited DC_50_ values of 1.14 nM in DOHH2 lymphoma cells and 2.70 nM in TMD8 lymphoma cells. Moreover, the authors propose a synergistic effect between IRAK4 degradation and PI3Kδ inhibition to explain PROTAC-13 superior anti-inflammatory activity.

Chen et al. reported the first XPO1-targeting PROTACs, comparing pomalidomide-based and benzamide-based CRBN ligands (Figure 5)^100^. SAR studies revealed that PEG linkers outperformed alkyl chains, and benzamide-based PROTACs showed greater efficacy than pomalidomide-based analogues with the same linker length. PROTAC-14 based on ligand compound 11 exhibited potent XPO1 degradation (DC_50_ = 23.67 nM) and strong antiproliferative activity against MV4-11 and MOLM-13 leukaemia cells (IC_50_ = 0.142 μM and 0.186 μM, respectively). In MV4-11 leukaemia cells, PROTAC-14 induced near-complete XPO1 degradation within 12h at 100 nM, along with caspase-mediated apoptosis, G1 phase arrest, nuclear factor kappa-light-chain-enhancer of activated B cells (NF-κB) inhibition, and reduced migration.

Jackson et al. report the discovery and optimisation of PROTAC-15 (CFT8634), a potent, selective, and orally bioavailable heterobifunctional degrader of BRD9 (Figure 5)^101^. Initial structure-activity relationship studies focused on replacing the phthalimide CRBN-binding moiety with an anilino glutarimide group (compound 12), which improved key physicochemical properties including reduced tPSA and decreased hydrogen bond acceptor count, thereby enhancing membrane permeability and oral absorption potential, despite an initial reduction in degradation potency (BRD9: DC_50_ = 160 nM). Subsequent linker optimisation through incorporation of a piperazine spacer significantly enhanced degradation efficacy (BRD9: DC_50_ = 5 nM). PROTAC-15 achieved potent BRD9 degradation (DC_50_ = 3 nM) with exceptional selectivity over BRD4 (DC_50_ > 10 µM), BRD7 (DC_50_ > 10 µM), and CRBN neosubstrates (IKZF1, GSPT1, SALL4. In BROMO scan profiling, PROTAC-15 (100 nM) engaged exclusively with BRD9 among tested bromodomains. Preclinical assessment revealed favourable pharmacokinetic properties with high oral bioavailability across species and demonstrated dose-dependent tumour growth inhibition in synovial sarcoma xenograft models.

Huang et al. identified PROTAC-16 (XYD270) (Figure 5) as a potent BRD9-targeting PROTAC with pronounced preclinical efficacy in synovial sarcoma (SS) and acute myeloid leukaemia (AML)^102^. Following optimisation of the BRD9 ligand and rigid linker, the authors focused on the CRBN-recruiting moiety and found that the compound 12 used in PROTAC-15 conferred only moderate BRD9 degradation, whereas removal of the aryl fluorine substituent to give the compound 13 markedly improved degradation potency. The optimised PROTAC-16 induced highly potent BRD9 degradation, with a DC_50_ of 0.082 nM and D_max_ of 96% in HS-SY-II cells, and a DC_50_ of 3.9 nM and D_max_ of 90% in MV4;11 cells; at 10 nM, it achieved 89% degradation within 0.5 h. PROTAC-16 also showed excellent selectivity, with no detectable degradation of BRD4, BRD7, or the CRBN neosubstrates IKZF1, GSPT1, and CK1α, and displayed potent antiproliferative activity, with IC_50_ values of 1.65 μM in HS-SY-II cells and 0.05 μM in MV4;11 cells. Collectively, these results demonstrate that fine-tuning of the CRBN ligand was critical for enhancing both BRD9 degradation and cellular activity.

Xie et al. developed a novel series of achiral CRBN ligands derived from substituted PDHU to overcome the limitations associated with racemic glutarimide-based CRBN ligands in PROTACs (Figure 5)^79^. Through systematic SAR analysis, they identified trisubstituted PDHUs – particularly compound 14 – that exhibited CRBN-binding affinities comparable to lenalidomide while displaying significantly improved stability under physiological and plasma conditions. Structural modelling revealed that optimised binding was driven by favourable hydrophobic contacts and a key hydrogen bond. To demonstrate functional utility, the authors linked PDHU to JQ1, generating several PROTACs. Among them, PROTAC-18 showed potent and selective BRD4 degradation across multiple cell lines, with a DC_50_ of 0.022 nM and D_max_ of 97% at 10 µM in MV4;11 leukaemia cells, alongside induction of apoptosis and G1 phase cell cycle arrest.

Yan et al. unexpectedly discovered that 3-aminophthalic acid compound 15 (OP-C-P1), a hydrolysis product of pomalidomide, functions as a CRBN ligand and developed it into an PROTAC targeting the ETS-related gene (ERG) transcription factor (Figure 5)^103^. Phthalic acid lacks the hydrolysis-prone imide ring, offering superior chemical stability and cost-effectiveness. PROTAC-19 degraded ERG with a DC_50_ of 172.4 nM in VCaP cells, comparable to pomalidomide-based analogues. Molecular docking revealed that compound 15 forms hydrogen bonds with key residues of CRBN. Mechanistic studies confirmed that degradation was CRBN-dependent and mediated via the ubiquitin-proteasome pathway. Functionally, PROTAC-19 suppressed ERG target gene expression, inhibited prostate cancer cell growth in 3D spheroid assays, and reduced cell invasion, highlighting its therapeutic potential.

### Tricyclic, spirocyclic, and fused heterocycles

While early CRBN ligands predominantly relied on relatively planar IMiD scaffolds, their limited three-dimensionality has increasingly been linked to suboptimal ligand efficiency, poor oral bioavailability, insufficient ternary complex cooperativity, and unintended neosubstrate degradation^25^^,^^104^. Accordingly, tricyclic, spirocyclic, and fused heterocyclic CRBN ligands have been developed to overcome these interconnected limitations.

By enforcing defined three-dimensional binding geometries and controllable linker exit vectors, these ligands enhance effective CRBN engagement, improve ternary complex formation, and markedly optimise pharmacokinetic properties, including oral bioavailability and metabolic stability. At the same time, rigidified scaffolds reduce off-target and neosubstrate-driven degradation, enabling the concurrent optimisation of affinity, bioavailability, selectivity, and in vivo efficacy.

**Figure 6.**
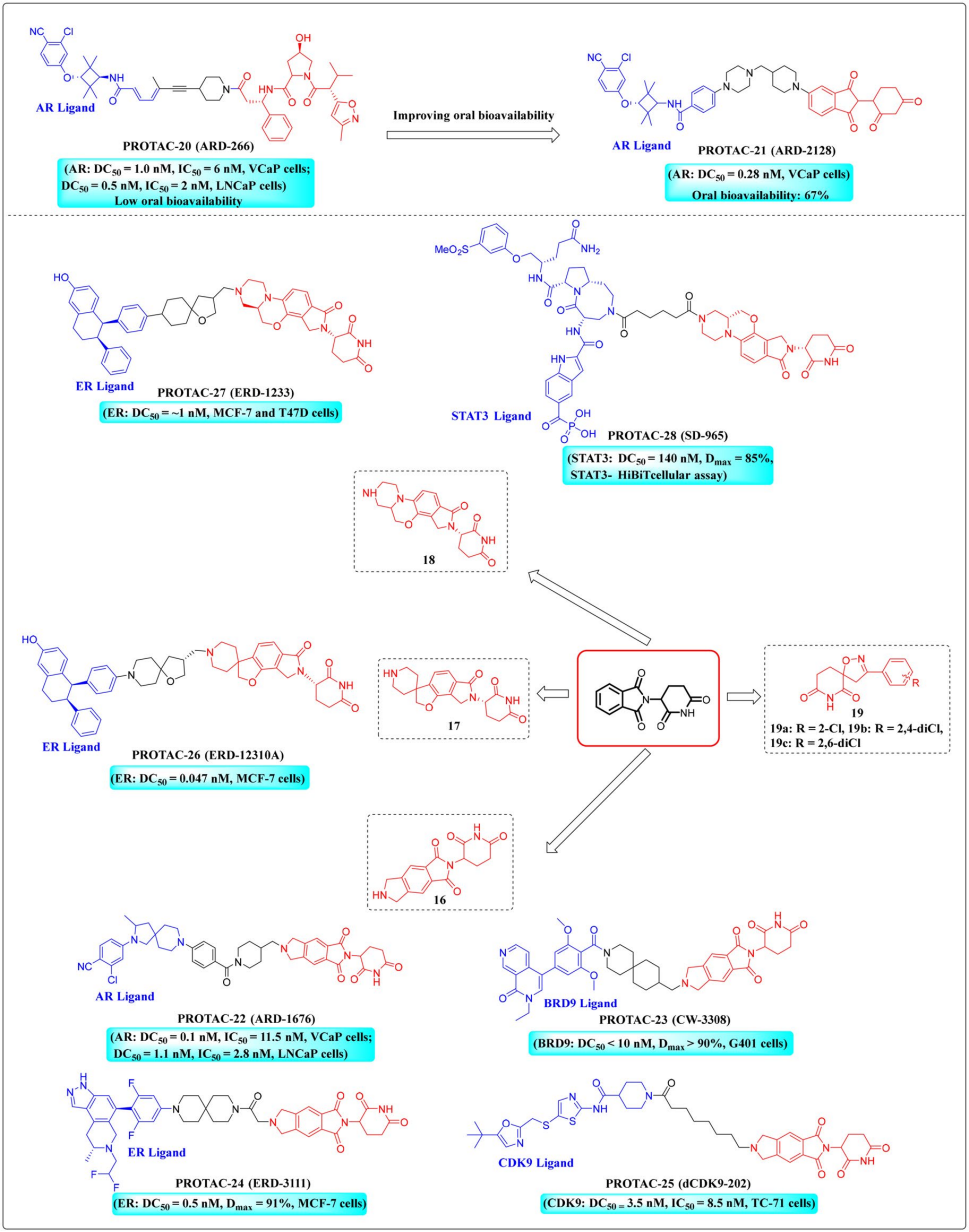
PROTACs based on tricyclic, spirocyclic and fused heterocyclic CRBN Ligands. Figure created using Chemdraw20.0 software.

Xiang et al. reported a novel CRBN-recruiting ligand, compound 16, derived through iterative optimisation. Cyclisation at the C5 and C6 positions of thalidomide yielded a tricyclic CRBN ligand, leading to the development of an orally bioavailable PROTAC degrader, PROTAC-22 (ARD-1676)^105^. Initially, von Hippel-Lindau (VHL)-based AR-PROTACs (e.g. ARD-61, ARD-69, ARD-266) exhibited poor oral bioavailability^48^^,^^106–109^. To address this limitation, Han et al employed aryloxy cyclobutylamine as an AR ligand and conjugated it with thalidomide via a piperazine linker, resulting in ARD-2128 and ARD-2585, which demonstrated 51% oral bioavailability and robust in *vivo* efficacy (Figure 6)^69^^,^^106^. Further structural modification of thalidomide led to the development of the CRBN ligand (compound 15), which was subsequently utilised to construct PROTAC-22. This PROTAC showed potent AR degradation (DC_50_ = 0.1 nM in VCaP, 1.1 nM in LNCaP) and antiproliferative activity (IC_50_ = 11.5 nM in VCaP, 2.8 nM in LNCaP), while degrading multiple clinically relevant AR mutants. Oral bioavailability was favourable across species – 67% (mice), 44% (rats), 31% (dogs), and 99% (monkeys). In VCaP xenografts, PROTAC-22 markedly reduced intratumoral AR and inhibited tumour growth without observed toxicity.

Wang et al. similarly conducted systematic optimisation by utilising the novel tricyclic CRBN ligand, compound 16, and successfully developed an efficacious and selective BRD9 PROTAC degrader, PROTAC-23 (CW-3308) (Figure 6)^110^. By modifying the BRD9-binding moiety and linker, PROTAC-23 was identified with excellent degradation potency (DC_50_ < 10 nM, D_max_ > 90%) and high selectivity over BRD7 and BRD4. It exhibited favourable oral bioavailability (91%) and strong in *vivo* efficacy, reducing BRD9 protein levels by > 90% in xenograft tumour tissues and achieving 60% tumour growth inhibition without toxicity. PROTAC-23 demonstrated superior antiproliferative activity in BRD9-dependent cancer cell lines, highlighting its potential for therapeutic development.

Chen et al. also employed the novel CRBN ligand, compound 16, which exhibited comparable binding affinity to thalidomide and lenalidomide along with superior absorption, distribution, metabolism, excretion (ADME) properties (Figure 6)^111^. Subsequently, through linker optimisation and incorporation of a tricyclic indazole ER ligand, they developed an efficacious ER degrader, PROTAC-24 (ERD-3111). PROTAC-24 achieved potent ERα degradation (DC_50_ = 0.5 nM, D_max_ = 91%). It exhibited favourable oral bioavailability (42% in mice, 20% in rats, 66% in dogs) and high tumour exposure. In *vivo*, PROTAC-24 effectively degraded wild-type and mutant ERα (Y537S, D538G) and induced strong tumour regression in MCF-7 xenograft models, performing comparably or superior to ARV-471 without observable toxicity.

Ma et al. developed the highly potent PROTAC cyclin-dependent kinase 9 (CDK9) degrader PROTAC-25 (dCDK9-202) by conjugating the CDK9 inhibitor SNS032 with a novel CRBN ligand (Figure 6)^112^. Compound 16 demonstrated exceptional degradation potency in TC-71 neuroectodermal tumour cells with a DC_50_ of 3.5 nM and achieving 94.4% CDK9 depletion at 25 nM. PROTAC-25 exhibited potent antiproliferative activity in TC-71 neuroectodermal tumour cells (IC_50_ = 8.5 nM) and consistently showed low nanomolar inhibitory effects across multiple cancer cell lines. In TC-71 xenograft models, intravenous administration (10 mg/kg) significantly suppressed tumour growth without observed toxicity.

Rej et al. substituted the amino group with a hydroxyl group in lenalidomide, which demonstrated a 3-fold improvement in CRBN binding affinity (Figure 6)^113^. Subsequently, by cyclizing the hydroxyl group with a benzene ring and introducing a spiro-piperidine moiety, compound 17 was generated. By coupling lasofoxifene with the novel CRBN ligand compound 16, which exhibited excellent binding affinity, a highly potent and orally active ERα PROTAC degrader, PROTAC-26 (ERD-12310A) with outstanding metabolic stability, was developed. Structure-guided linker optimisation led to PROTAC-26 with a DC_50_ of 0.047 nM in MCF-7 mammary cancer cells, significantly outperforming ARV-471. The compound effectively degraded both wild-type and ESR1Y537S mutant ERα *in vitro* and in *vivo*. Oral administration resulted in favourable PK profiles and robust antitumor efficacy, achieving tumour regression in ER wild-type and strong inhibition in ESR1-mutant xenograft models without detectable toxicity.

Acharyya et al. designed PROTAC-27 (ERD-1233) by conjugating lasofoxifene with a novel CRBN ligand compound 18 (RR-11055) (Figure 6)^114^. Compound 18 was obtained by introducing a piperazine ring and forming a cyclized structure. This ligand exhibits interactions with key residues of CRBN, demonstrating excellent binding affinity (IC_50_ = 0.38 μM) and favourable ADME properties. Through systematic linker optimisation, PROTAC-27 achieved potent ERα degradation in MCF-7 and T47D mammary cancer cells (DC_50_ ≈ 1 nM). Oral administration in mice and rats demonstrated favourable pharmacokinetics and significant antitumor efficacy, including 68% tumour regression in the MCF-7 xenograft model and effective suppression of ESR1 Y537S mutant tumours.

Wang et al. developed PROTAC-28 (SD-965), a potent and selective signal transducer and activator of transcription 3 (STAT3) degrader, through optimisation of the STAT3 ligand, linker, and CRBN ligand (Figure 6)^115^. In the optimisation of the CRBN ligand, the team started from the high-affinity ligand compound 18 and systematically evaluated a series of novel CRBN ligands, including tricyclic, spiro, benzimidazolone, and indazole derivatives. Based on comprehensive pharmacokinetic and in *vivo* efficacy profiles, PROTAC-28 was identified as the most promising candidate. In a STAT3-HiBiT cellular assay, PROTAC-28 potently induced STAT3 degradation with a DC_50_ of 0.14 μM and a D_max_ of 85%. In MOLM-16 leukaemia and SU-DHL-1 lymphoma cells, PROTAC-28 exhibited IC_50_ values of 1.3 nM and 260 nM, respectively, demonstrating superior antiproliferative activity compared to other reported STAT3 degraders. Global proteomics analysis confirmed its high selectivity for STAT3 among over 7,000 proteins. Pharmacokinetic studies showed that PROTAC-28 had a moderate half-life of 1.44 h, slow clearance (496 ml/h/kg), and high plasma exposure (AUC_0-24h_ = 3976 h·ng/mL) in mice (2 mg/kg, IV), and exhibited excellent metabolic stability across species (t₁/₂ > 120 min in both plasma and microsomes).

Shevalev et al. designed a series of spiro-isoxazole-based glutarimide derivatives as novel CRBN ligands (Figure 6)^116^. Incorporation of the spirocyclic isoxazole motif introduced a unique binding conformation and exit vector within the CRBN pocket, thereby improving metabolic stability and minimising off-target degradation. Several compounds, notably, showed enhanced CRBN affinity (*K*_i_ = 3.6–3.8 μM) compared to thalidomide (*K*_i_ = 8.5 μM), with favourable physicochemical properties. Among them, compound 19a also demonstrated improved plasma stability (*t*_1/2_ = 298 min). In addition, compound 19b exhibited low cytotoxicity in myeloma cells and peripheral blood mononuclear cells (PBMCs).

### C-terminal cyclic imide (cyclimid)

Although IMiD-based CRBN ligands provide robust E3 recruitment, their intrinsic molecular glue activity and limited tuneability often lead to neosubstrate engagement and restricted linker design flexibility. To overcome these constraints, C-terminal cyclic imide (Cyclimid) CRBN ligands were developed as a modular platform that preserves the essential CRBN-binding pharmacophore while enabling systematic structural diversification^117^.

Cyclimids replace the rigid phthalimide core with customisable cyclic imide motifs, allowing fine-tuning of steric environment, exit vector orientation, and physicochemical properties. This design strategy enables improved control over ternary complex formation and target selectivity, while significantly reducing unintended neosubstrate degradation. As a result, Cyclimids represent a chemically versatile and mechanistically distinct class of CRBN ligands, which have been reported in the literature^118^.

**Figure 7.**
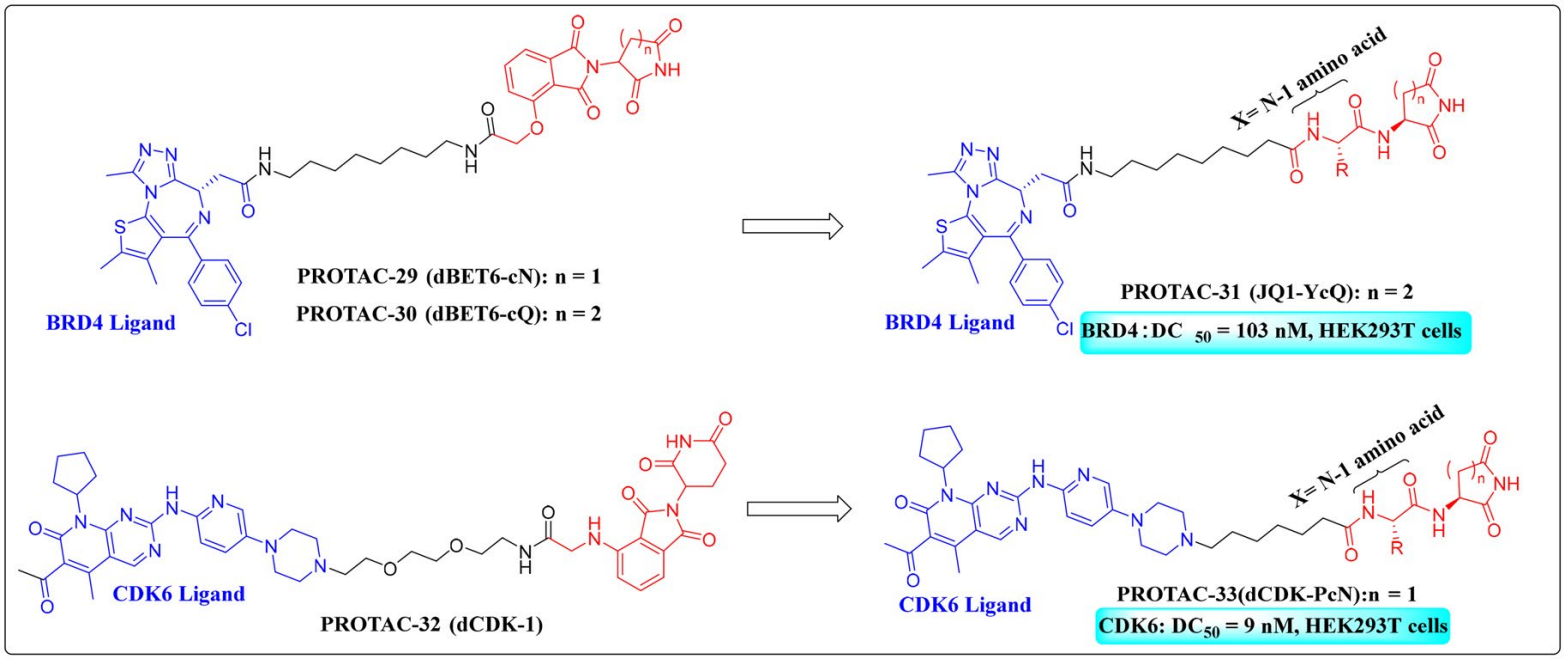
PROTACs based on C-terminal cyclic imide. (General representation of C-terminal cyclic-imide degrons (XcQ and XcN). X denotes any of the 20 canonical amino acids at the N-1 position; R represents the peptide backbone or N-terminal extension. X = Y (Tyrosine) and X = P (Proline)). Figure created using Chemdraw20.0 software.

Ichikawa et al. developed a series of CRBN-recruiting ligands, termed cyclimids, derived from C-terminal cyclic imide degrons^119^. By incorporating 5-membered aspartimide (cN) or 6-membered glutarimide (cQ) motifs and coupling to JQ1 (Figure 7), FKBP, or CDK-targeting moieties, over 60 bifunctional degraders were synthesised. time-resolved fluorescence resonance energy transfer (TR-FRET) assays showed that cN-based cyclimids bound CRBN with higher affinity (*K*_D_ = 8 nM) than cQ analogs or thalidomide derivatives. These degraders formed distinct ternary complexes with enhanced cooperativity and exhibited potent cellular degradation of BRD4 (e.g. PROTAC-31 (JQ1-YcQ), DC_50_ = 103 nM), FK506-binding protein 12 (FKBP12), and CDK6 (e.g. PROTAC-33 (dCDK-PcN), DC_50_ = 9 nM) in HEK293T kidney cells. Cyclimid-based degraders also minimised off-target degradation of neosubstrates such as IKZF3 and GSPT1, demonstrating favourable selectivity and drug-like properties.

### Covalent CRBN ligands

The emergence of covalent targeted protein degradation strategies aims to overcome the limitations of traditional reversible degraders, such as suboptimal degradation efficiency and transient engagement, which result from their short-lived target binding^120^. The core of this approach lies in using covalent bonds to irreversibly stabilise key interactions within the degradation complex, thereby extending its lifetime and enhancing degradation efficacy^121^. Two primary pathways exist to achieve this goal: covalent modification of the POI or covalent modification of the E3 ligase. Among these, the development of covalent E3 ligase ligands has garnered significant attention in recent years, as it offers a potential “platform-based” solution. Once a potent and selective covalent E3 ligand is obtained, it can be linked to various target protein binders to construct durable covalent PROTACs, enabling robust degradation of multiple targets^122^.

Among E3 ligases, CRBN stands out as an ideal candidate for rational covalent ligand design due to the presence of multiple cysteine residues on its molecular surface that are accessible to electrophilic warheads such as acrylamides and chloroacetamides^123^. This structural feature provides a direct foundation for developing covalent CRBN ligands, which – through precise targeting of these residues – may overcome the substrate limitations of traditional ligands, expand the degradation scope, and improve both degradation efficiency and selectivity^124^.

Building on this rationale, covalent CRBN ligands have progressed from concept to practical applications. For example, Cruite et al. designed EM12-based covalent probes equipped with sulphonyl fluoride or fluorosulfate warheads to target His353 within the CRBN binding pocket^125^. Their studies demonstrated that EM12-SO₂F covalently labels His353, effectively inhibits CRBN activity, and blocks lenalidomide-induced IKZF1 degradation. Meanwhile, EM12-FS retained selectivity while inducing selective degradation of the neo-substrate N-terminal glutamine amidohydrolase 1 (NTAQ1) via CRL4^CRBN^- and neddylation-dependent mechanisms. These findings illustrate that covalent modification of CRBN can not only achieve effective inhibition but also enable programming of novel degradation profiles through fine-tuning of the warhead. Furthermore, this strategy has been successfully extended to PROTAC design.

**Figure 8.**
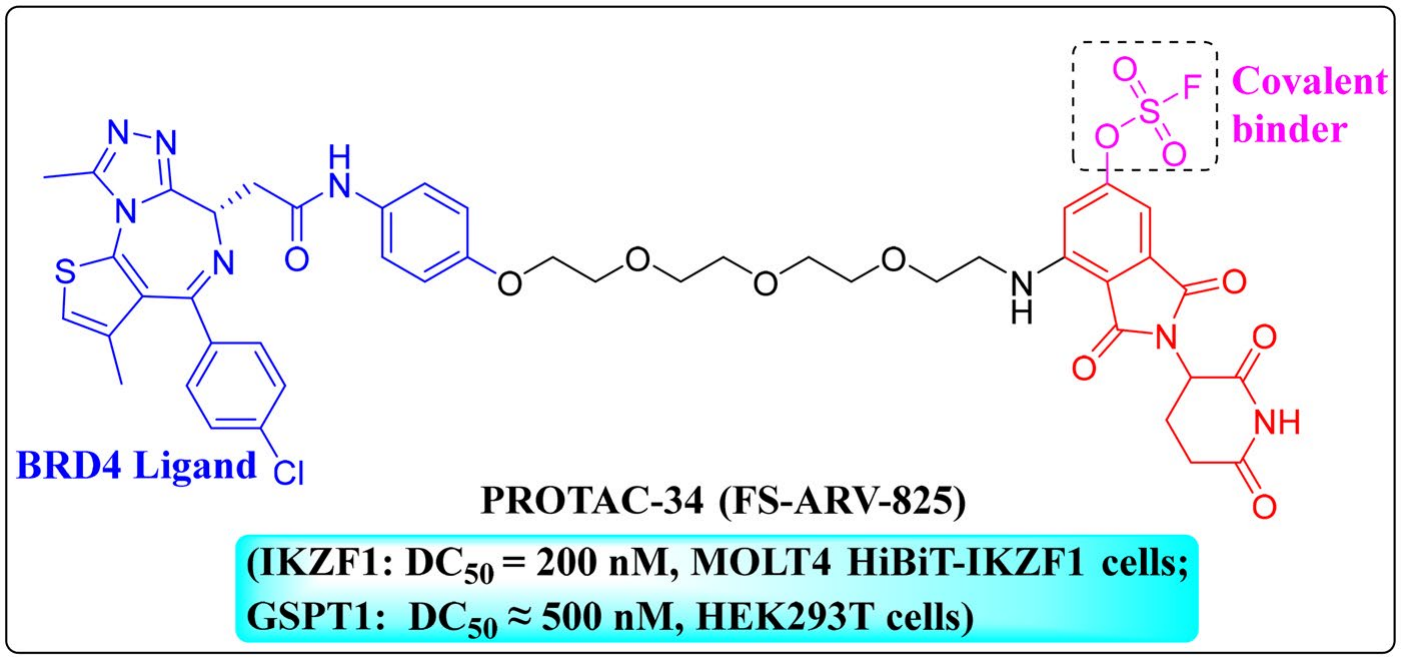
PROTAC based on covalent CRBN ligand. Figure created using Chemdraw20.0 software.

Nowak and Jones et al. developed PROTAC-34 (FS-ARV-825), a covalent CRBN-based PROTAC incorporating a fluorosulfate warhead at the 6-position of the phthalimide ring to target His353 in CRBN (Figure 8)^126^. Mass spectrometry confirmed ∼50% covalent labelling of CRBN. PROTAC-34 effectively degraded BRD4 in HEK293T kidney cells with sustained activity after compound washout, and induced degradation of BRD2/3/4 in MOLT4 leukaemia cells. Compared to PROTAC-2 (ARV-825), PROTAC-34 showed reduced activity towards neosubstrates IKZF1 (DC_50_ = 200 nM) and GSPT1 (DC_50_ ≈ 500 nM). The compound exhibited favourable plasma and hepatocyte stability, highlighting its potential for covalent PROTAC design.

### Hypoxia-activated CRBN ligands

A major limitation of current CRBN-based PROTACs is the lack of spatial selectivity, leading to systemic degradation of target proteins in both diseased and healthy tissues. To mitigate this issue, hypoxia-activated CRBN ligands have been developed to exploit the distinct microenvironmental features of solid tumours^127^.

These ligands remain inactive under normoxic conditions but undergo chemical or enzymatic activation in hypoxic environments, thereby selectively enabling CRBN recruitment and target degradation in tumour tissues^128^. By integrating environmental responsiveness into CRBN ligand design, this strategy introduces an additional layer of spatiotemporal control, improving therapeutic index and reducing off-target toxicity.

**Figure 9.**
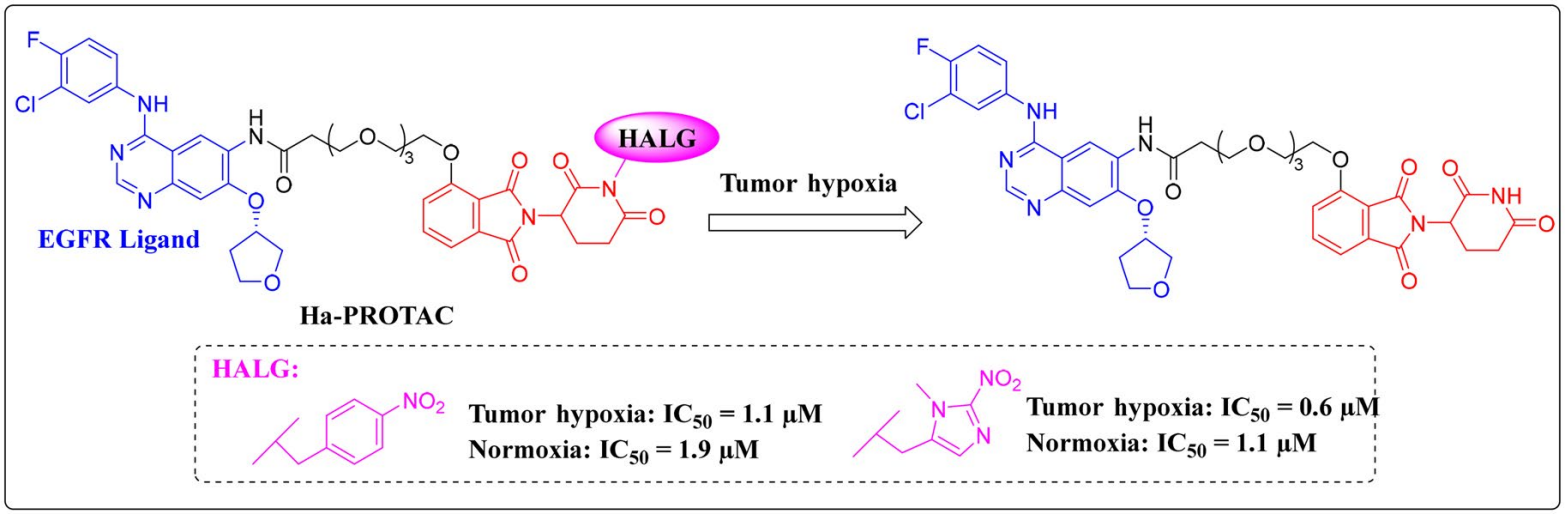
ha-PROTACs based on hypoxia-activated CRBN ligands. Figure created using Chemdraw20.0 software.

Cheng et al. developed two hypoxia-activated hypoxia-activated PROTACs (ha-PROTACs) by introducing hypoxia-sensitive moieties into the CRBN ligand, thereby enhancing target selectivity (Figure 9)^129^. Both ha-PROTACs selectively degraded epidermal growth factor receptor deletion in exon 19 (EGFR Del19) under hypoxic conditions in PC9 lung cancer cells. Ligands bearing nitropyrazole hypoxic groups showed better hypoxia selectivity, with minimal degradation in normoxia. In cell viability assays, these two ligands exhibited enhanced potency under hypoxia (IC_50_ = 1.09 and 0.61 μM) compared to normoxia (IC_50_ = 1.92 and 1.1 μM). Both ha-PROTACs also inhibited cell migration and induced apoptosis more effectively under hypoxia.

Overall, recent CRBN ligand optimisation strategies reflect a shift from IMiD-based modification towards structure-guided engineering of CRBN recruiters. Through the development of non-IMiD scaffolds, rigid three-dimensional architectures, programmable cyclimid motifs, and stimulus-responsive designs, the major mechanistic limitations of classical IMiDs have been progressively addressed, resulting in improved ternary complex cooperativity, selectivity, and pharmacokinetic performance (Table 2).

**Table 2.** Comparative summary of next-generation CRBN ligand classes.

Ligand class	Representative scaffold	Key advantages	Major limitations	Oral bioavailability	Stability	Neosubstrate degradation/selectivity
Non-IMiD	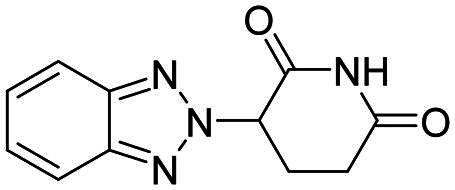	Decoupled IKZF1/3 degradation, improved PK, lower tPSA	Affinity sometimes weaker	Improved	Improved hydrolysis resistance	Reduced off-target
Benzamid/ PDHU	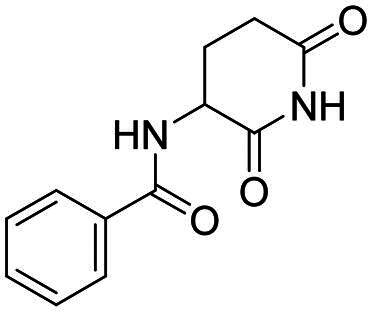	Improved chemical stability	Less molecular glue activity	/	/	Reduced GSPT1 degradation, programmable selectivity
Tricyclic/ Spirocyclic	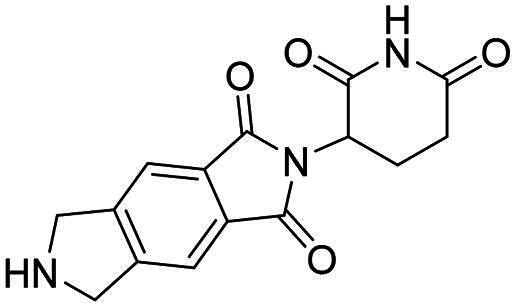	Enhanced ternary cooperativity, improved PK properties	/	Improved	Rigidified geometry	/
Cyclimids	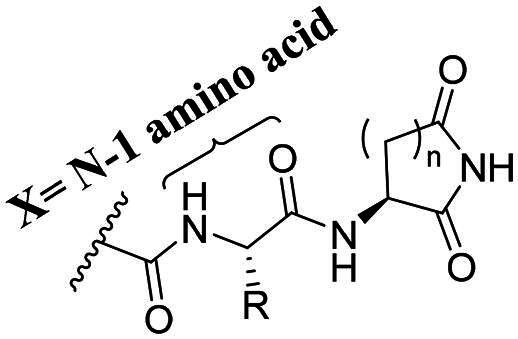	Tuneable degron recognition, modular design	/	/	Improved	Minimal IKZF3/GSPT1 degradation
Covalent CRBN ligands	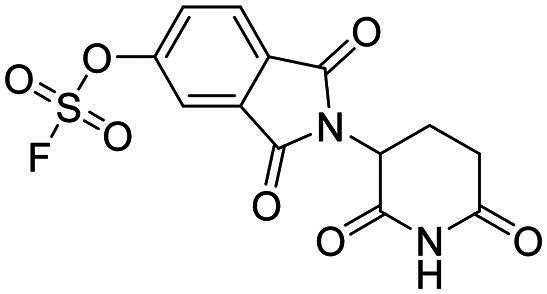	Sustained degradation, prolonged residence time, irreversible engagement	Safety concerns, warhead optimisation required	/	Improved	/
Hypoxia-activated	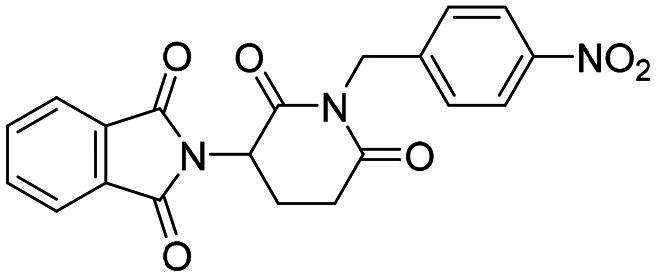	Spatially controlled degradation, tumour selectivity	Activation efficiency limited validation	/	/	Improved tumour selectivity

The chemical innovations described above have not only expanded the toolbox for PROTAC design but also provided a fertile ground for the rational development of CRBN-dependent molecular glue degraders. Unlike classical IMiDs, whose neosubstrate selectivity was largely discovered serendipitously, next-generation CRBN ligands with defined binding geometries and tuneable surface topologies offer the potential to program molecular glue activity in a predictable manner. As discussed in the following section, this paradigm shift is beginning to enable the targeted degradation of previously inaccessible neo-substrates such as GSPT1, CK1α, and ZBTB11.

## Beyond PROTACs: CRBN ligands in molecular glue degraders

**Figure 10.**
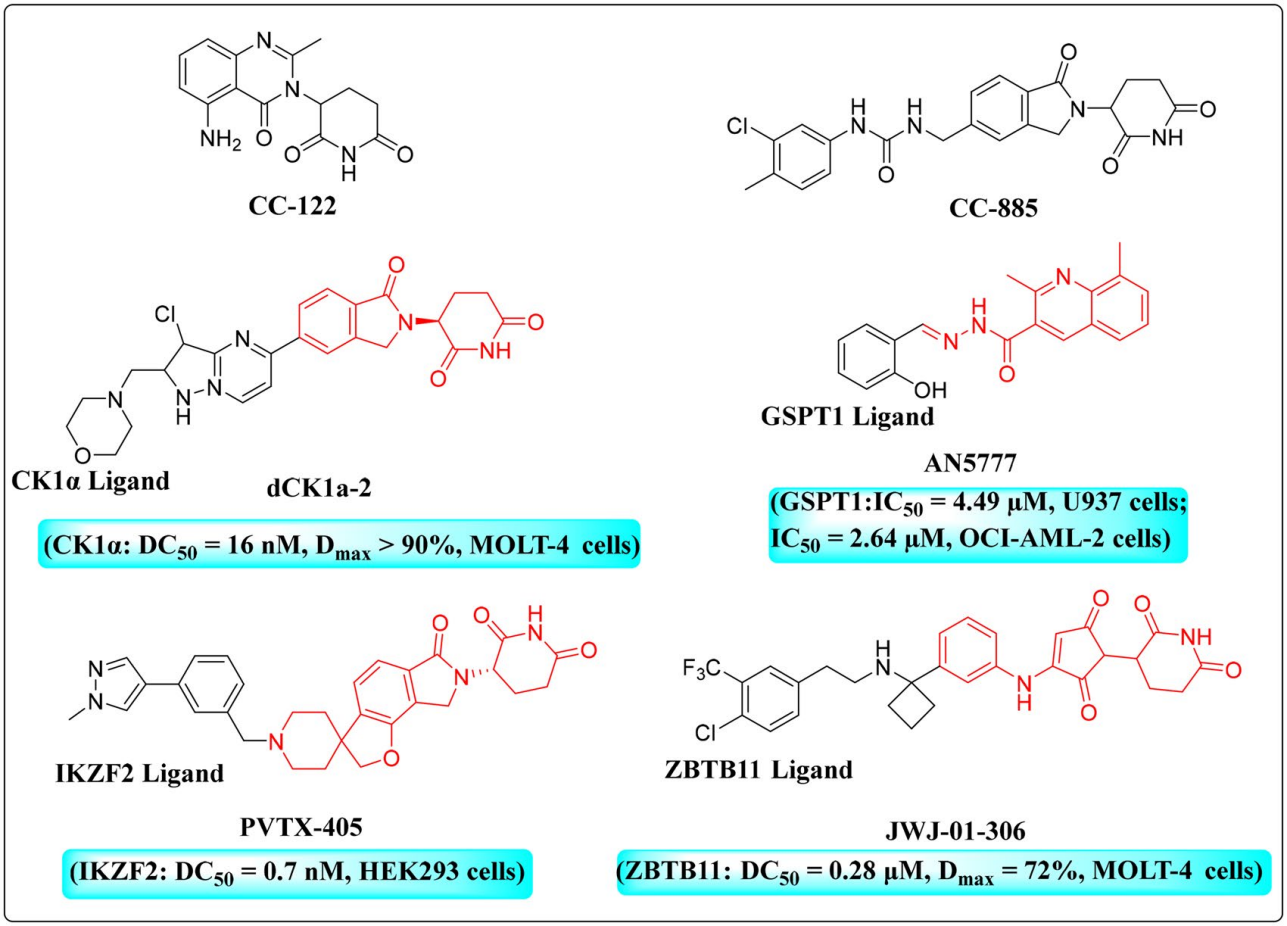
CRBN-recruiting molecular glue degraders. Figure created using Chemdraw20.0 software.

Building upon the classical IMiD paradigm of zinc finger–directed degradation, next-generation analogues such as CC-122 (avadomide) (Figure 10) exemplified incremental optimisation within this framework, retaining IKZF1/3 degradation capability while enhancing CRBN engagement and pharmacokinetic properties^68^^,^^130^. These refinements demonstrated that subtle scaffold modifications can improve potency without fundamentally altering substrate specificity. A more pronounced shift emerged with CC-885 (Figure 10), in which C4 substitution on the IMiD core redirected substrate preference towards the translation termination factor GSPT1, representing the first clear example of substrate reprogramming within the CRBN glue landscape.^19^ Structural analyses of CRBN–IMiD–neosubstrate ternary complexes further substantiated this mechanistic reprogramming model, demonstrating that subtle ligand modifications remodel the CRBN substrate-binding interface and thereby dictate neosubstrate selection^19^^,^^25^. This finding revealed that minimal chemical perturbations are sufficient to reshape the CRBN surface topology and alter neosubstrate compatibility.

Huang et al. rationally designed dCK1α-2 as an orally bioavailable and selective CK1α molecular glue degrader through systematic structure-activity relationship optimisation (Figure 10)^131^. Shifting the isodindolone attachment from the 3- to the 6-position of the pyrazolo [1,5-a] pyrimidine core with a chlorine substituent at the R1 position enhanced degradation potency, while incorporation of a morpholine group into the solvent-exposed region improved solubility and pharmacokinetic properties. dCK1α-2 induced potent CK1α degradation with DC**_50_** of 16 nM and D_max_ > 90% in MOLT4 HiBiT-CK1α cells, demonstrating excellent selectivity over IKZF2 and GSPT1. Mechanistic studies confirmed CRBN-dependent ternary complex formation and proteasomal degradation, leading to p53 pathway activation. dCK1α-2 exhibited favourable oral bioavailability (*F* = 34%) and robust in vivo efficacy, achieving 55% tumour growth inhibition at 20 mg/kg in MOLM13 xenograft models without observable toxicity.

While traditional CRBN molecular glues are largely confined to the chemical and functional space of IMiDs, their scope and selectivity are inherently limited by this scaffold^104^. The emergence of novel CRBN ligand chemotypes (e.g. tricyclic, spirocyclic, benzamide) breaks this constraint. By remodelling the CRBN binding interface, these ligands enable the programmable design of new molecular interaction surfaces.^91^ This expanded chemical toolkit is thus pivotal not only for advancing heterobifunctional PROTACs but also for the rational development of a new class of CRBN-dependent molecular glue degraders, moving beyond serendipitous discovery towards engineered substrate targeting^10^.

Zhang et al. reported the discovery of non-IMiD GSPT1 molecular glue degraders through a structure-guided virtual screening and bioassay workflow (Figure 10)^132^. Substructure-based optimisation established clear structure-activity relationships, leading to AN5777 as the most potent analog. AN5777 showed potent antiproliferative activity against U937 (IC_50_ ≈ 4.49 μM) and OCI-AML-2 cells (IC_50_ = 2.64 μM). Functionally, AN5777 induced G1 phase arrest and apoptosis in leukaemia cells.

Chen et al. developed a novel CRBN molecular glue degrader, PVTX-405 (Figure 10)^133^. PVTX-405 demonstrated excellent degradation potency and selectivity (DC_50_ = 0.7 nM in HEK293 cells, with minimal off-target degradation of other CRBN neosubstrates such as IKZF1/3 and SALL4, along with enhanced safety and pharmacokinetic profiles. In the MC38 syngeneic tumour model, PVTX-405, either as a monotherapy or in combination with immune checkpoint inhibitors, effectively inhibited tumour growth and prolonged animal survival.

Jiang et al. reported the discovery and optimisation of JWJ-01–306, a first-in-class CRBN-recruiting molecular glue degrader targeting ZBTB11 (Figure 10)^134^. Guided by Rosetta-based ternary complex modelling, systematic SAR exploration identified cyclobutyl substitution and 4-chloro-3-trifluoromethyl left-hand aryl modification as key drivers for improved degradation efficacy. The optimised compound JWJ-01–306 achieved potent zinc finger and BTB domain-containing protein 11 (ZBTB11) degradation (DC₅。 = 0.28 μM, D_max_ = 72%) with improved selectivity over IKZF1. The cellular thermal shift assay (CETSA) confirmed CRBN-dependent ternary complex formation, and proteomics revealed selective ZBTB11 degradation with minimal off-targets. In dabrafenib-resistant melanoma models, JWJ-01–306 suppressed oxidative phosphorylation and inhibited proliferation, validating ZBTB11 as a metabolic vulnerability to overcome BRAF inhibitor resistance.

Importantly, beyond the classical IMiD framework, emerging CRBN ligand chemotypes – such as tricyclic, spirocyclic, and benzamide-based scaffolds – are now being actively explored for molecular glue applications. For instance, the tricyclic CRBN ligand used in ARD-1676 (PROTAC-22) not only improves oral bioavailability but also remodels the CRBN surface in a manner that could favour selective engagement of alternative neo-substrates. Similarly, the PDHU scaffold, which intrinsically lacks the molecular glue activity of IMiDs, provides a ‘clean’ starting point for installing glue function through rational side-chain engineering. These advances suggest that the boundary between PROTACs and molecular glue degraders is becoming increasingly blurred, with both approaches benefiting from the same next-generation CRBN ligand chemistry.

## Conclusions

PROTACs represent a catalytic, event-driven alternative to occupancy-based inhibitors, enabling the degradation of traditionally undruggable targets^135^. Over 30 candidates are currently in clinical trials^136–139^. However, their typically high molecular weight, flexibility, and polar surface area often result in poor solubility, permeability, and oral bioavailability^138^^,^^140^. To address these limitations, rational design strategies – such as linker rigidification, reduction of hydrogen-bond donors/acceptors, and optimisation of ternary complex cooperativity – are being employed to improve metabolic stability, cell permeability, and overall pharmacokinetic profiles without compromising degradation efficacy^17^^,^^138^^,^^141^. These constraints have shifted increasing attention towards the rational optimisation of E3 ligase ligands as a decisive leverage point for improving the developability of degraders.

Among the repertoire of E3 ligase recruiters, CRBN has emerged as the most tractable and widely utilised scaffold, owing to its well-characterised binding pocket and the robust clinical precedent established by IMiDs^13^. However, the inherent limitations of first-generation CRBN ligands – such as off-target degradation of neosubstrates (e.g. IKZF1/3), immunomodulatory side effects, and metabolic instability^104^^,^^142^, and emerging drug resistance driven by CRBN mutations, target alterations, or UPS dysfunction – have motivated the search for safer and more durable alternatives.

In response, the field has witnessed systematic evolution of CRBN ligands, spanning non-IMiD scaffolds, tricyclic and spirocyclic architectures, cyclimids, covalent binders, and stimuli-responsive designs. These structural innovations have enabled more precise exit-vector control, enhanced ternary complex cooperativity, reduced off-target degradation, and markedly improved pharmacokinetic profiles including oral bioavailability. Moreover, chemically diverse CRBN ligands have emerged as fertile starting points for the development of molecular glue degraders, enabling selective and context-dependent degradation of previously inaccessible substrates such as GSPT1 and IKZF2.

Despite these advances, several critical challenges remain unresolved. Looking forward, four key directions are likely to shape the next phase of CRBN-based degrader development. First, drug resistance – whether through CRBN mutations, target alterations, or UPS dysfunction – requires next-generation ligands that accommodate CRBN mutants, together with multi-target degraders or combination strategies. Second, substrate selectivity demands deeper mechanistic insight into ternary complex formation and neosubstrate recognition, where integration of structural biology, biophysics, and chemoproteomics will be essential^143–145^. Third, oral bioavailability can be further advanced through linker rigidification, reduction of hydrogen-bond donors/acceptors, and conformationally constrained scaffolds (e.g. tricyclic, spirocyclic, and PDHU-based designs). Fourth, the increasing application of artificial intelligence (AI) and structure-guided computational approaches is expected to accelerate both PROTAC and molecular glue degrader discovery by enabling predictive modelling of ternary complex stability and cooperativity, thereby reducing empirical trial-and-error optimisation^146^^,^^147^.

In summary, the continuous chemical innovation of CRBN ligands has transformed CRBN from a convenient E3 recruiter into a programmable degradation hub. By bridging PROTACs and molecular glue degraders, next-generation CRBN ligands are redefining the boundaries of targeted protein degradation. Ongoing efforts to integrate ligand chemistry, structural insight, and systems-level biology will be pivotal in translating CRBN-based degraders into safer, more selective, and clinically durable therapies for complex human diseases.

## Data Availability

Data sharing is not applicable to this article, as no new data were created or analysed in this study.
